# *TNFAIP3*/A20 dysfunction drives innate and sterile hyperinflammation

**DOI:** 10.3389/fimmu.2026.1856810

**Published:** 2026-07-17

**Authors:** Karel F. A. Van Damme, Pieter Hertens, Dorine Sichien, Katrien Van der Borght, Justine Van Moorleghem, Sofie De Prijck, Alex Klarenbeek, Els Louagie, Inés Lammens, Stijn Vanhee, Christian Vanhove, Pieter De Bleser, Steven Van Laecke, Amélie Dendooven, Hamida Hammad, Lars Vereecke, Dirk Elewaut, Geert van Loo, Bart N. Lambrecht

**Affiliations:** 1 Laboratory of Mucosal Immunology, VIB-UGent Center for Inflammation Research, Ghent, Belgium; 2 Department of Internal Medicine and Pediatrics, Faculty of Medicine and Health Sciences, Ghent University, Ghent, Belgium; 3 Department of Rheumatology, Ghent University Hospital, Ghent University., Ghent, Belgium; 4 Laboratory of Cellular and Molecular (Patho)physiology, VIB-UGent Center for Inflammation Research, Ghent, Belgium; 5 Department of Biomedical Molecular Biology, Ghent University, Ghent, Belgium; 6 argenx, Ghent, Belgium; 7 VIB Flow Core, VIB Center for Inflammation Research, Ghent, Belgium; 8 Upper Airways Research Laboratory, Department of Head and Skin, Ghent University, Ghent, Belgium; 9 IBiTech—Medisip—Infinity Lab, Ghent University, Ghent, Belgium; 10 Data Mining and Modeling for Biomedicine Group, VIB-UGent Center for Inflammation Research, Ghent, Belgium; 11 Renal Division, Department of Internal Medicine, Ghent University Hospital, Ghent, Belgium; 12 Division of Pathology, University Hospital Ghent, Ghent, Belgium; 13 Host-Microbiota-Interaction Laboratory, VIB-UGent Center for Inflammation Research, Ghent, Belgium; 14 Cancer Research Institute Ghent, Ghent University, Ghent, Belgium; 15 Molecular Immunology and Inflammation Laboratory, VIB-UGent Center for Inflammation Research, Ghent, Belgium; 16 Ghent Gut Inflammation Group, Ghent University, Ghent, Belgium; 17 Department of Pulmonary Medicine, Erasmus MC, Rotterdam, Netherlands; 18 Department of Respiratory Medicine, Ghent University Hospital, Ghent, Belgium

**Keywords:** A20, B cells, germ-free, innate immunity, lupus, TNFAIP3

## Abstract

Feedback mechanisms regulate immune activation and prevent excessive tissue damage. TNFAIP3, also known as A20, serves as a crucial brake on inflammation, and mutations or haploinsufficiency of this gene are linked to diseases characterized by inappropriate inflammation. In this study, we document highly conserved patterns of cell type-specific gene expression, regulation, and induction of *TNFAIP3*, and employ transgenic and gnotobiotic mouse models to investigate how adaptive immunity and the gut microbiome contribute to pathology arising from impaired A20 function. Contrary to our expectations, systemic inflammation resulting from *Tnfaip3* deficiency in CD11c (*Itgax*)-expressing cells developed independently of autoreactive antibodies, B cells, and T cells. The microbiome also proved dispensable for disease manifestations in these models. These findings suggest that in diseases caused by insufficient TNFAIP3/A20 activity, autoantibodies may reflect a downstream consequence of disease rather than a causative driver, suggesting autoinflammatory rather than autoimmune pathology. These insights carry therapeutic implications for the treatment of *TNFAIP3*-associated diseases.

## 1 Introduction

**Figure 1:**
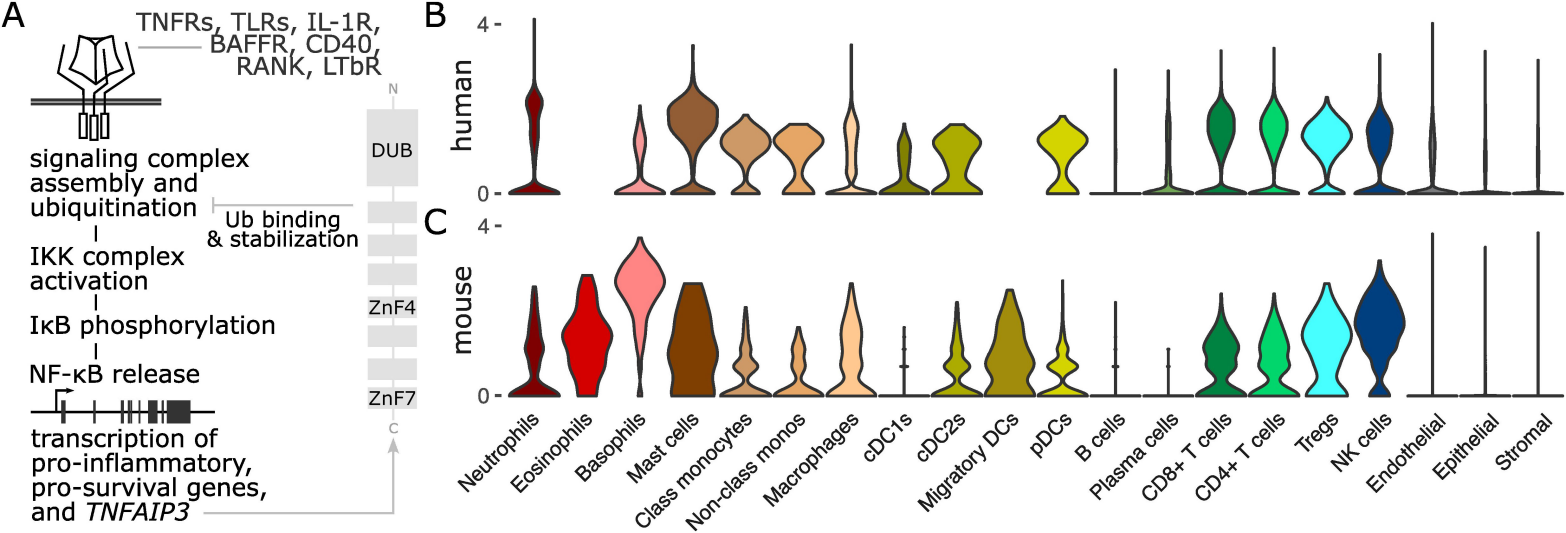
*TNFAIP3*/A20 function **(A)** and transcription in humans **(B)** and mice **(C)**.

Feedback mechanisms are critical to fine-tune immune responses, ensuring that inflammation does not result in disproportionate tissue injury. A20, initially discovered as a tumour necrosis factor (TNF)-induced protein 3 (TNFAIP3) (1), plays an essential role to control the extent and duration of inflammation. Upon ligand binding to receptors such as the TNF receptor, interleukin-1 receptor, B or T-cell receptor, CD40, receptor activator of NF-κB (RANK), or pattern recognition receptors such as Toll-like receptors (TLRs) and NOD-like receptors (NLRs), an intracellular signaling cascade results in the activation of nuclear factor kappa-light-chain enhancer of activated B cells (NF-κB) (2, 3) (**Figure 1A**). NF-κB induces the transcription of genes involved in cell survival and inflammation in a cell type- and tissue-specific manner. To limit excessive inflammation and prevent cell death, NF-κB induces the expression of *TNFAIP3* (4). Through its ubiquitin (Ub)-binding domains, A20 interacts with ubiquitinated proteins to terminate sustained NF-κB signaling and inhibit cell death (5–9).

The importance of *Tnfaip3* in maintaining immune homeostasis was first observed in full-body A20-knockout mice, which develop severe cachexia and organ-wide inflammation, resulting in premature mortality (10). Cell type and context-dependent functions of A20 have been identified using conditional genetic knockout and experimental models. For example, myeloid-specific A20-deficient mice spontaneously develop polyarthritis resembling rheumatoid arthritis (RA), driven by NLRP3 inflammasome-mediated macrophage necroptosis (9, 11, 12). Similarly, mice with dendritic cell (DC)-specific A20 knockout exhibit multi-organ inflammation with lymphosplenomegaly, myeloid expansion, and spontaneous lymphocyte activation (13), resembling systemic lupus erythematosus (SLE) (14) or inflammatory bowel disease (IBD) (15). In B cells, A20 is required for their normal differentiation and to prevent the formation of autoantibodies and glomerular immune complex formation (16–18). Furthermore, T cell and NK cell-targeting strategies established *Tnfaip3* as an inherent checkpoint during lymphocyte development, activation, and survival (19–22).

In line with these experimental findings, numerous studies identified polymorphisms in the *TNFAIP3* locus linked to autoimmune, autoinflammatory, and allergic disorders (23, 24). *TNFAIP3* variants have been identified in patients with conditions such SLE, RA, psoriasis, IBD, juvenile idiopathic arthritis, systemic sclerosis, type 1 diabetes mellitus, multiple sclerosis, Sjögren’s disease, and celiac disease (3, 25). Conversely, *TNFAIP3* variants may also confer increased resistance to infections (26, 27). Many of these single nucleotide polymorphisms (SNP) are enriched in genomic regions involved in regulating *TNFAIP3* transcription (25). Beyond non-coding variants, loss-of-function mutations in *TNFAIP3* have been identified in patients with a Behçet-like disease, featuring oral ulcers, fever, mucosal and skin involvement, autoimmunity, and arthritis (28). This disease is inherited in an autosomal dominant fashion and has been termed A20 haploinsufficiency (HA20) (29, 30). These associations underline the critical importance of feedback by A20 in maintaining immune homeostasis.

Given the clinical impact of *TNFAIP3* dysregulation, we first established a data-driven framework describing its expression, regulation, and induction across species. The strong conservation between humans and mice prompted us to investigate universal drivers of *TNFAIP3*-associated disorders in mice. Because autoantibodies are a hallmark of many immune-mediated disorders, and they are frequently detected in patients with HA20 (30–32), we tested their contribution to systemic inflammation in mice with a CD11c Cre-driven loss of *Tnfaip3*. Furthermore, given prior evidence linking the microbiome to immune dysregulation (33–36), we examined whether the microbiome alters systemic inflammation in these mice.

## 2 Materials and methods

### 2.1 Mouse housing, genetics, and rederivation

Experiments were performed with a mixture of male and female mice, unless explicitly mentioned otherwise. All animals were maintained at specific-pathogen-free conditions in individually ventilated cages with 12-hour day/night cycles. Food and water was provided ad libitum for the duration of the experiments, unless described differently. All *in vivo* experimental procedures were approved by the animal ethical committee of the VIB Center for Inflammation Research.

All experiments were performed on mice of C57Bl/6 genetic background. Conditional A20/*Tnfaip3* knockout mice, in which exons 4 and 5 of the *Tnfaip3* gene are flanked by two LoxP sites, were generated as described before (37) and crossed to mice expressing a CD11c-driven Cre recombinase (38). These animals are hereafter referred to as A20^CD11c-ko^ mice hereafter. To evaluate the role of IgGs, B cells and all lymphocytes, we further crossbred these mice to homozygous *Fcgrt*-deficient (39), *Ighm*-deficient (‘µMt’) (40), and a *RAG2*-deficient (41) backgrounds, respectively. For breeding, due to infertility of A20^CD11c-ko^ mice, homozygous *Tnfaip3*-floxed Cre-negative females were cohoused with heterozygous *Tnfaip3*-floxed Cre-expressing males. To evaluate the targeting by the *Itgax* Cre, these mice were interbred with mice expressing TdTomato in the Rosa26 locus preceded by a floxed stop codon (42).

Germ-free or axenic mice were generated by embryo transfer in axenic recipients at the germ-free mouse facility of the University of Ghent. Axenic mice were housed under positive-pressure flexible film isolators (North Kent Plastics).

### 2.2 Analysis of single-cell RNA-sequencing datasets

*TNFAIP3* expression was evaluated in the Tabula Sapiens (43), non-immune cells in the Tabula Muris (44), and mouse splenocytes as described previously (45). The regulatory potential calculation of transcription factors on *TNFAIP3* was carried out as described previously (45). Cytokines affecting *TNFAIP3* expression in human cells were retrieved from CytoSig (46). Cytokines represented by at least three independent datasets and significantly associated with altered TNFAIP3 expression were selected using a Wilcoxon signed-rank test. The top 15 cytokines were subsequently visualized as previously described (45).

The *in vivo* effect of cytokines on *Tnfaip3* expression was assessed in the Immune Dictionary (47). We extracted the annotation as provided, experimental group, and *Tnfaip3* expression for each cell from an integrated Seurat object (https://singlecell.broadinstitute.org/single_cell/study/SCP2554/dictionary-of-immune-responses-to-cytokines-at-single-cell-resolution) in R v4.4.0. We calculated the average *Tnfaip3* (LogNormalized) RNA counts for each cytokine and cell type (with at least 3 cells). Per cell type and cytokine, Log_2_ fold changes and *p* values (Wilcoxon test) were calculated in comparison to the PBS (control) group and filtered if *p* < 0.05 (Suppl. File 2). Cytokines which modulated *Tnfaip3* expression in > 1 population were retained and their Log_2_ fold changes per cell population and cytokine were plotted using ComplexHeatmap v2.20.0.

### 2.3 GWAS studies

The GWAS catalog v1.0.2 was downloaded on October 15th, 2025 from https://www.ebi.ac.uk/gwas/docs/file-downloads (48). All SNPs associated with the gene(s) of interested, including both reported and mapped genes, were retained. Non-disease-related associations were excludes. The number of unique SNPs, identified by their RSIDs, was summarized and plotted per association.

### 2.4 Flow cytometry

All organs were isolated following terminal bleeding and kept on ice until processing. Spleens were cut into small pieces and digested in RPMI containing 2% fetal bovine serum (FBS), 20 µg/mL Liberase (Roche; 05 401 119 001) and 10 U/mL DNAse I (Roche; DN2) at 37 °C for 30’. Following centrifugation, cells were passed through a 70 µm filter (Corning; 431751) and red blood cell lysis was carried out on ice for 3 minutes.

To generate single-cell suspensions of the bone marrow, the tibial bone was flushed out with RPMI through a 70 µm filter, followed by an osmotic lysis step for 30 seconds on ice. The Peyer’s patches were passed through a nylon mesh with 70 µm pores to generate a single cell suspension, which was followed by a washing step.

For the isolation of immune cells from the intestinal lamina propria, the distal 5.4 cm of the colon was opened, cleaned roughly in cold PBS with curved tweezers, cut into small segments of 0.5 x 0.5 cm, and stored in 2% FBS/RPMI on ice until further processing. Tissues were washed twice with pre-warmed RPMI and then incubated twice for 20 minutes in 2 mM EDTA/RPMI in a shaking water bath at 37 °C, with a washing step in between. Next, cells were enzymatically digested using collagenase VIII (Sigma; 2139) 1 mg/mL and DNase at 1/2000 for approximately 30 minutes at 37 °C in a shaking warm water bath. After digestion, cells were passed through a 70 µm cell strainer, suspended in 2% FBS/RPMI on ice until staining.

10^4^ counting beads (eBioscience cat. 01-1234-42) were added to each well and the cells were stained with fluorescently labeled antibodies and Fc block (kind gift from Louis Boon (JJP Biologics), clone 2.4G2) during 30 min at 4 °C. Where applicable, intracellular staining was carried out in a second phase after fixation and permeabilization (Invitrogen; 00-5523-00).

Samples were measured on a BD LSRFortessa or BD FACSymphony A5. Downstream analysis was performed in Flowjo v10.9.0 (BD). Gating was carried out as displayed in **Supplementary Figure 4**. The populations not shown were identified as follows: cDC1s as XCR1^+^ cDCs; cDC2s as CD172a^+^ cDCs; eosinophils as SiglecF^hi^ SSC-A^hi^; γδ T cells as CD3e^+^ CD90^+^ CD4^-^ CD8a^-^ γδTCR^+^; ILCs as CD90^hi^ CD3e^neg^; pDCs as Siglec H^+^ BST2^+^; germinal center B cells as GL7^+^ CD95^+^ B cells; plasma cells as CD138^+^ CD43^+^ or CD138^+^ IgD^-^.

### 2.5 CITE-sequencing on wild-type and A20^CD11c-ko^ splenocytes

3 A20^CD11c-ko^ mice and 3 littermate *Tnfaip3*^fl/fl^ *Itgax*-Cre^-^ mice were included for single cell sequencing, all female and 12 weeks old. Mice were injected IV with pentobarbital containing fluorescently labeled anti-CD45 antibodies to exclude circulating cells. To facilitate cell sorting, we carried out magnetic-activated cell sorting (MACS) on enzymatically digested splenocytes to remove neutrophils, B and T cells from a portion of the splenocytes. Briefly, 80x10^6^ splenocytes were stained with FITC-labeled CD3, CD19 and Ly-6G antibodies and Fc block for 30’. Following washing, 160 µL anti-FITC beads (Miltenyi Biotec; 130-048-701) were added for 15’ at 4 °C. The resuspended cells were then passed through a LS column (Miltenyi Biotec; 130-042-401) in a magnetic field and the effluent was collected. All samples were manually counted and 4x10^6^ cells were isolated and spun down. The cell pellet was resuspended and incubated for 30’ on ice with 50 µL of staining mix in PBS containing 0.04% BSA, fluorescently labeled antibodies, Fc block, TruStain FcX Block (BioLegend; 101320), the mouse cell surface protein antibody panel containing 160 oligo-conjugated antibodies and 9 TotalSeq-A isotype controls (TotalSeq-A, BioLegend) and TotalSeq-A cell hashing antibodies (BioLegend) diluted 1:1000. 35000 viable cells (a fixed number of CD45^-^, CD19^+^, CD3^+^, Ly6G^+^, CD11c or 120G8^+^ and residual CD45^+^ cells) were sorted per organ in BSA-coated tubes on FACSAria II and III (BD Biosciences). All antibody details are included in Supplementary File 5.

Sorted single-cell suspensions were resuspended at an estimated final concentration of 1000 cells/µl and loaded on a Chromium GemCode Single Cell Instrument (10x Genomics) to generate single-cell gel beads-in-emulsion (GEM). Biological replicates were multiplexed per lane. The scRNA-Seq libraries were prepared using the GemCode Single Cell 3’ Gel Bead and Library kit, version 3 (10x Genomics) according to the manufacturer’s instructions with the addition of amplification primers (3nM each, 5’CCTTGGCACCCGAGAATT*C*C - 5’GTGACTGGAGTTCAGACGTGTGC*T*C) during cDNA amplification to enrich the TotalSeq-A cell surface and hashing protein oligos respectively. Size selection with SPRIselect Reagent Kit (Beckman Coulter; B23318) was used to separate amplified cDNA molecules for 3’ gene expression and cell surface protein construction. TotalSeq-A protein library construction including sample index PCR using Illumina’s TruSeq Small RNA primer sets and SPRIselect size selection was performed according to the manufacturer’s instructions. The cDNA content of pre-fragmentation and post-sample index PCR samples was analyzed using the 2100 BioAnalyzer (Agilent). Sequencing libraries were loaded on an Illumina NovaSeq flow cell at VIB Nucleomics core with sequencing settings according to the recommendations of 10x Genomics, pooled in a 70:20:10 ratio for the combined 3’ gene expression, cell surface and hashing protein samples, respectively.

The Cell Ranger pipeline (10x Genomics, version 6.1.2) was used to perform sample demultiplexing and to generate FASTQ files for read 1, read 2 and the i7 sample index for the gene expression, cell surface and hashing protein libraries. Read 2 of the gene expression libraries was mapped to the mouse reference genome (GRCm38.99). Subsequent barcode processing, unique molecular identifiers filtering and gene counting was performed using the Cell Ranger suite. CITE-seq reads were quantified using the feature-barcoding functionality. The average of the mean reads per cell across all gene expression libraries was 22500, with an average sequencing saturation of 44%, as calculated by Cell Ranger.

For each sample individually, the Cell Ranger count matrices were transformed into SeuratObjects with Seurat. The HTO, ADT and RNA data were integrated. Doublets were removed with the DoubletFinder v2.0.3 package and merged in Seurtt. Cells with extreme parameters were removed (≥ 50000 RNA reads, ≤ 200 or ≥ 6000 genes, ≤ 5 or ≥ 140 ADTs detected, ≤ 10 or ≥ 6000 ADT reads, or ≥ 10% mitochondrial reads). The HTO and ADT counts were normalized via centered log transformation and scaled. The RNA counts were log normalized. The PCAs were calculated for both RNA and ADT data using the general Seurat workflow. A weighted nearest neighbor graph was created in Seurat based on the first 40 PCAs of RNA and first 20 PCAs of ADT data (49). We clustered at a resolution of 1 (smart local moving algorithm), which resulted in a good separation between the major cell populations. We determined differentially expressed genes and proteins and annotated clusters manually. Further subsetting was carried out on T and NK cells, and on conventional dendritic cells to enable a more fine-grained annotation.

Data wrangling and visualization was carried out using tidyverse v2.0.0, viridis v0.6.5, ggpubr v0.6.0, scales v1.3.0. Identification of biological replicates was based on the highest number of hashing antibodies. Expression levels of Tnfaip3 were min-max scaled for visualization purposes. Differentially expressed genes were calculated using the default FindMarkers function in Seurat. Genes expressed in at least 10% of the population, with an absolute log2 fold-change of > 0.5, and an adjusted p value< 0.05 (Wilcoxon Rank Sum test) were used as input for pathfindR v2.4.1 to evaluate enrichment of Mus musculus KEGG pathways. Additional pathway analyses were performed using gene sets retrieved from msigdbr v7.5.1. For each pathway, per-cell module scores were calculated using the default Seurat AddModuleScore function. Statistical differences in pathway activity between conditions were assessed using a Wilcoxon rank-sum test.

### 2.6 ELISAs

Serum immunoglobulins were measured by enzyme-linked immunosorbent assay (ELISA) in serum in half area microplates (Greiner; 675061). Wells were incubated with 50 µL of coating antibodies overnight at 4 °C and blocked with 150 µL 1% casein (Merck; C7078) in PBS for two hours. Samples were diluted in 0.1% casein buffer (IgA: 1/200; IgM: 1/3200; IgG: 1/30000). 50 µL of samples or standards were incubated for 2 hours at RT on an orbital shaker. Afterwards, detection antibodies were added for 1 hour. Mouse IL-6 (Invitrogen; 88-7064) and TNF (Invitrogen;. 88-7324) were measured by ELISA, according to the manufacturer’s instructions. B-cell activating factor (BAFF) was quantified on ELISA (R&D; DY2106-05) according the manufacturer’s instructions. Mouse anti-dsDNA IgG or IgA were quantified on ELISA (Alpha Diagnostic; 5120) according to the manufacturers’ instructions. For the measurement of anti-cardiolipin antibodies, microplates (Greiner; 675061) were coated with 50 μg/mL cardiolipin from bovine heart (Sigma; C1649) in 100% ethanol. Following overnight incubation at room temperature, plates were blocked with 1% bovine serum albumin in PBS. After serum incubation for 2 h, HRP-labeled goat anti-mouse IgG or IgA (Southern Biotec; 1030–05 or 1040-05) was added for 1 h. For all ELISAs, following thorough washing, 50 µL development substrate (Invitrogen; 00-4201-56) was added and the reaction was stopped using 25 µL 1.25 M sulphuric acid (VWR; 20700.298). The absorbance for each sample was measured at 450 and 650 nm.

### 2.7 Imaging

The kidneys were freshly frozen in OCT (Sakura; 4583) and stored at −80 °C. Cryosections of 10 to 20 μm thickness were fixed for 2 to 10’ in 2% PFA at room temperature. Sections were blocked for 60’ with 1% goat and 1% rat serum for immunoglobulin detection, and with 1% rat serum for complement detection. Following washing, primary labeled antibodies and DAPI (ThermoFisher; D21490) were added for 2 h. Slides were mounted with polyvinyl alcohol (Sigma; 10981), and imaged on a laser scanning microscope (Zeiss LSM-780). Quantification of fluorescent intensity was carried out in ImageJ. Z stacks were compressed at maximal intensity, the glomerular area were manually marked and the mean fluorescent intensity was measured per glomerular area for each fluorophore. The mean of 3 to 5 representative glomeruli per mouse was plotted.

Kidneys were placed overnight in 4% paraformaldehyde and embedded in paraffin for light microscopy. Sections were made at 3 μm thickness, manually stained with hematoxylin and eosin or periodic acid-Schiff (PAS), and imaged using a Zeiss AxioScan Z1.

### 2.8 Quantitative PCR evaluation

Whole spleen was homogenized with a TissueLyser (Qiagen) and further processed for RNA extraction using TRIzol Reagent (Invitrogen; 10296-010) according to the manufacturer’s instructions. RNA content was measured on a NanoDrop Spectrophotometer (Thermo Scientific) and 1 μg RNA was transferred for cDNA conversion using the sensiFAST cDNA synthesis kit (Biolin; 65054). cDNA of interest was amplified by 30 cycles of PCR with sensiFAST SYBR No-ROX kit (Bioline; 98050) on a LightCycler 480 system (Roche). The following primers were used: *Igha1* (AGTGCACAGTTACCCATCCTG, AGGGACACGAGCTCATTCAG), *Il17a* (GCTCCAGAAGGCCCTCAGACT, CCAGCTTTCCCTCCGCATTGA), *Reg3g* (GCTTCCCCGTATAACCATCA, GCATCTTTCTTGGCAACTTCA) and reference genes *Tbp* (TCTACCGTGAATCTTGGCTGTAAA, TTCTCATGATGACTGCAGCAAA), *Hprt* (TCCTCCTCAGACCGCTTT, CCTGGTTCATCATCGCTAATC), *Actb* (GCTTCTAGGCGGACTGTTACTGA, GCCATGCCAATGTTGTCTCTTAT), *Gapdh* (TGGCAACAATCTCCACTTTGC, ACAAAATGGTGAAGGTCGGTG). Analysis was carried out in qbase+ (Biogazelle) and Calibrated Normalized Relative Quantities (CNRQs) values were exported and normalized for the wild-type SPF control group.

### 2.9 PET-CT

PET-CT imaging was performed at the INFINITY lab of University Ghent as previously described (50). All animals were fasting for at least 6 h prior to imaging. Mice were shortly anesthetized using isoflurane for intravenous injection of 10 MBq of ^18^FDG dissolved in 200 µl 0.9% NaCl. Directly after tracer injection, mice were awoken and rested in their cages. 30’ after tracer injection, the animals were placed under general anesthesia using an isoflurane mixture (5% induction, 1.5% maintenance, 0.3 l/min) and a 15-min total-body PET scan was acquired on a dedicated small animal PET scanner (B-Cube, Molecubes, Belgium). Animals were placed in prone position, receiving further anesthesia through a nose cone. Body temperature was maintained at 37 °C. Each PET scan was followed by a total-body spiral CT scan (X-Cube, Molecubes). The acquired PET data were iteratively reconstructed into a 192 × 192 × 384 matrix with 400 µm isotropic voxel size. CT data were iteratively reconstructed into a 200 × 200 × 550 matrix with 200 µm isotropic voxel size. PET-CT images were processed and analyzed via Amide software. Standardized uptake values (SUV) were calculated to account for body weight and injected dose as follows:


$$\text{SUV}=\frac{\text{activity concentration}\left(\frac{\text{Bq}}{\text{mL}}\right)}{\text{injected dose}\left(\text{Bq}\right)/\text{body weight}\left(\text{g}\right)}.$$

### 2.10 Additional readouts

Fertility testing: Females with the tested genotype were co-housed with Cre-negative males carrying no, one, or two *Tnfaip3*-floxed alleles. The number of pups born were counted once weekly during 10 weeks.

Hematology tests: Complete blood counts, including measurement of hemoglobin levels and thrombocyte count were measured on blood in EDTA-coated tubes using an automated hematology analyzer at Ghent University Hospital.

Sedimentation: Erythrocyte sedimentation rate was determined on 200 µL blood in citrate-buffered tubes (Sarstedt; 18.3125), which were left on the bench at room temperature (21 °C).

### 2.11 Statistical testing

GraphPad Prism V8 software or R v4.4.0 was used for statistical analysis. Results were expressed as the mean (± standard error of the mean), with each data point representing a biologically independent replicate. Statistical analysis between experimental groups was carried out using Mann–Whitney U tests (in case of two groups), t tests (for MFI values) with Benjamini-Hochberg correction, or two-way ANOVAs with Šídák’s correction for multiple testing.

## 3 Results

### 3.1 *TNFAIP3*/A20 regulation is highly conserved across species

Given the pivotal role of TNFAIP3/A20 in controlling inflammation, we sought to create a detailed overview of its expression across cell types and species. Using the Tabula Sapiens dataset, which maps gene transcription across human organs at single-cell resolution (43), we found that *TNFAIP3* is highly expressed in a large number of immune cells, including granulocytes, myeloid cells, T cells, and NK cells, whereas non-immune cells transcribed little *TNFAIP3* in healthy patients (**Figure 1B**). To profile *Tnfaip3* expression in mice, we analyzed splenocytes of steady-state mice which we described previously (45), and non-immune cells from the Tabula Muris (44). Consistent with its central role in immunoregulation, *Tnfaip3* exhibited a highly preserved expression profile across species in steady state (**Figure 1C**).

*TNFAIP3* expression is strongly induced in response to NF-κB activity (51). To further investigate its gene regulation, we calculated the ‘regulatory potential’ of transcription factors (52) binding within 10 kB of the *TNFAIP3* transcription start site as demonstrated by chromatin immunoprecipitation-seq experiments (53). In both humans and in mice, the p65/RELA subunit of NF-κB displayed the highest regulatory potential over *TNFAIP3* expression (**Supplementary Figure 1A**, **Supplementary Table 1**). Numerous additional transcription factors displayed regulatory potential of *TNFAIP3* both in humans and mice.

We extended this analysis by exploring upstream ligands that can induce or repress *TNFAIP3* expression. In humans, ligands from the TNF (TWEAK, CD40L, TNF, TRAIL) and IL-1 (IL-1α, IL-1β, IL-33) families were found to strongly upregulate *TNFAIP3* transcription based on the CytoSig database (46) (**Supplementary Figure 1B**). The Immune Dictionary single-cell dataset (47) provided more insights into murine *in vivo* and cell type-specific regulation of *Tnfaip3*. Myeloid cells as well as regulatory T cells strongly upregulated *Tnfaip3* in response to TNF (**Supplementary Figure 1C**, **Supplementary Table 2**). Interestingly, IL-3, IL-15, IL-18, and G-CSF increased *Tnfaip3* expression in monocytes, but repressed its transcription in other cell types. Overall, this comparative analysis indicates a conserved regulation of TNFAIP3, in line with previous reports on A20-deficient mice which phenocopy human diseases linked to A20 dysfunction (11, 14, 15).

### 3.2 A20 deficiency in CD11c-expressing cells drives systemic, multi-organ inflammation

**Figure 2:**
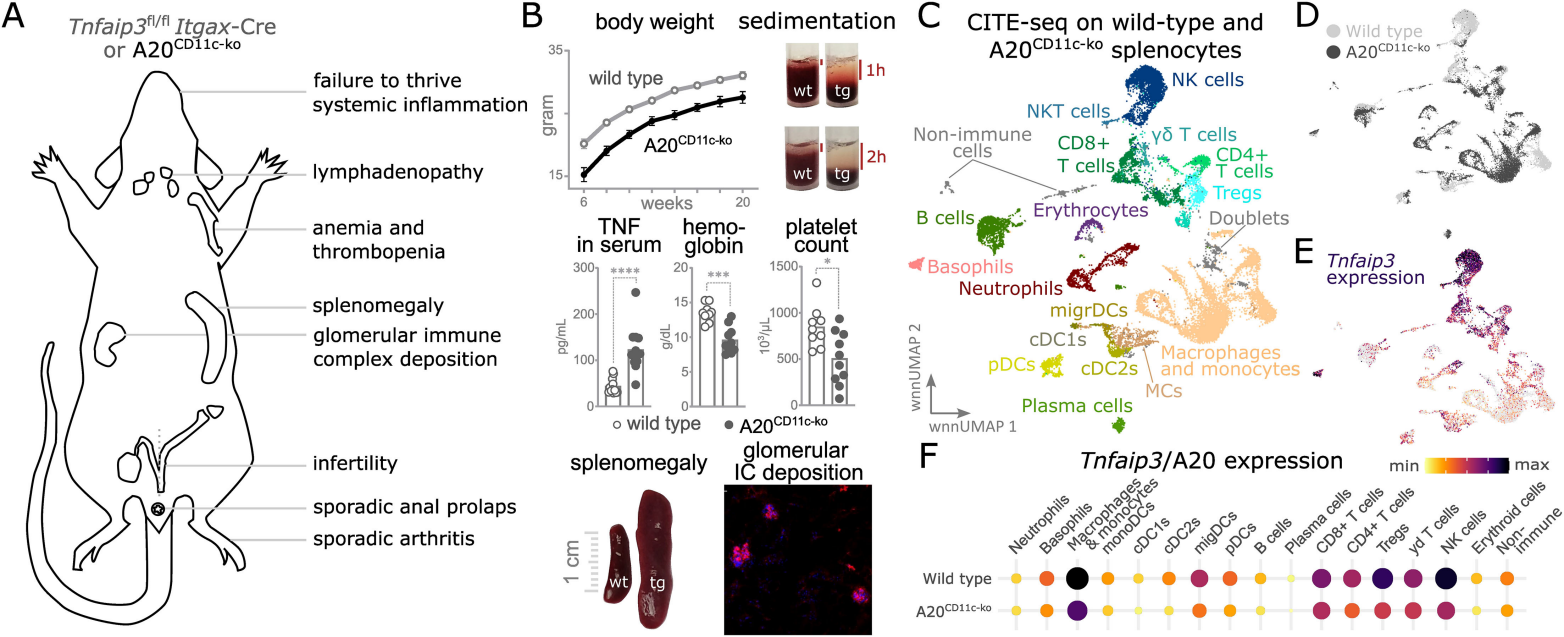
Systemic and multiomics evaluation of A20^CD11c-ko^ mice. **(A, B)** Disease manifestations in A20^CD11c-ko^ mice (body weight measurements: wild-type, n = 38; A20^CD11c-ko^, n = 27). **(C, D)** Weighted Nearest Neighbor Uniform Manifold Approximation and Projection (wnnUMAP) of splenocytes of A20^CD11c-ko^ mice and wild-type littermates **(C)** and colored according to genotype. **(E, F)** *Tnfaip3* expression overlaid on wnnUMAP **(E)** or shown as heatmap **(F)**. Error bars represent the standard error of the mean (body weight); *p < 0.05; ***p < 0.001; ****p < 0.0001.

Given the high accordance of A20 across species, we investigated mice with *Itgax* (CD11c)-driven knockout of *Tnfaip3* (A20^CD11c-ko^) to probe general disease mechanisms underlying A20 dysfunction. We specifically chose this model over other conditional *Tnfaip3*-deficient models (11, 16, 17, 27, 54), since they develop severe inflammation across multiple organ systems, characterized by weight loss, anemia, thrombocytopenia, lymphosplenomegaly, and glomerular immune complex deposition (**Figures 2A, B**) (14, 15). To gain an unbiased understanding of their immune dysregulation, we performed a Cellular Indexing by Transcriptomes and Epitopes (CITE)-sequencing experiment on sorted splenocytes from wild-type and A20^CD11c-ko^ mice (**Figures 2C, D**, **Supplementary Figures S2A-D**). A20^CD11c-ko^ mice exhibited marked NK cell lymphopenia, a loss of conventional dendritic cells, and a significant expansion of monocyte-derived cells (**Supplementary Figure 2E**) (browse the data online at: www.single-cell.be/data-explorer/browser/861b2647-304e-4280-ae67-e27772dbcf63). Despite increased serum levels of TNF in A20^CD11c-ko^ mice, *Tnfaip3* expression was consistently reduced across all examined cell populations, extending beyond CD11c^+^ dendritic cells (**Figures 2E, F**). Using reporter mice, we indeed confirmed that the CD11c-Cre targets 47.5% of all splenic immune cells in steady state (**Supplementary Figure 2F**). *Itgax*-driven recombination was highly efficient in cDC1s, cDC2s, pDCs as expected, but also affected almost all splenic macrophages and around half of B cells, T cells and NK cells (**Supplementary Figure 2F**). This suggests that the *Itgax*-driven approach resembles broad A20 dysfunction in immune cells, rather than a dendritic cell-restricted model (14, 15).

### 3.3 FcRn deficiency reduces immune complex deposition but fails to rescue systemic inflammation in CD11c-driven A20 deficiency

**Figure 3:**
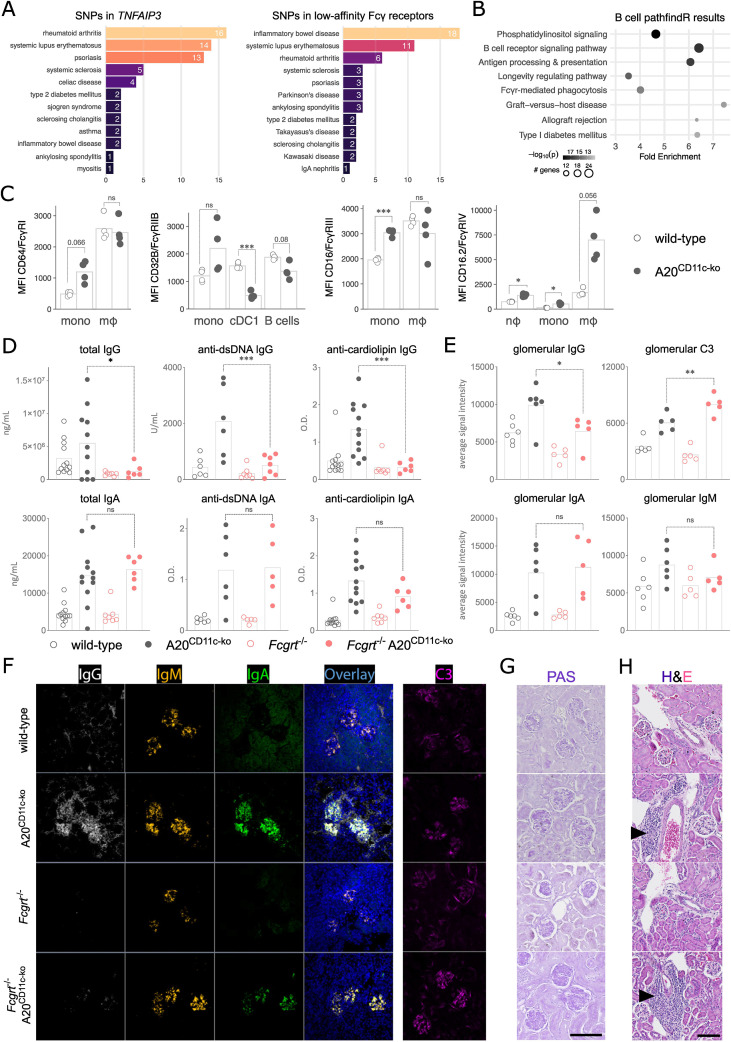
Antibodies and their effector functions in *TNFAIP3*-driven diseases and in A20^CD11c-ko^ mice. **(A)** The number of unique single nucleotide polymorphisms mapping to *TNFAIP3* or the low-affinity Fcγ receptor locus, according to the NHGRI-EBI GWAS Catalog. **(B)** Enrichment analysis on splenic B cells from A20^CD11c-ko^ versus wild-type mice. **(C)** Geometric mean fluorescent intensities (MFIs) of Fcγ receptor surface expression on splenocytes. **(D)** Serum immunoglobulin levels in wild-type, A20^CD11c-ko^, *Fcgrt*^-/-^, and *Fcgrt*^-/-^ A20^CD11c-ko^ mice. **(E)** Glomerular deposition of immunoglobulins and C3. **(F)** Representative confocal images by genotype. **(G-H)** Light microscopy of glomeruli in wild-type, A20^CD11c-ko^, *Fcgrt*^-/-^, and *Fcgrt*^-/-^ A20^CD11c-ko^ mice, stained with periodic acid-Schiff **(G)** or hematoxylin and eosin **(H)**. Black triangles point towards infiltrates and the scale bars indicate 50 µm. Statistical analyses were performed using t tests adjusted with Benjamini-Hochberg correction **(C)** and two-way ANOVAs with Šídák’s multiple comparison testing **(D-E)**. ns = non-significant; *p < 0.05; **p < 0.01; ***p < 0.001.

Because multiple single nucleotide polymorphisms (SNPs) near *TNFAIP3* and within the low-affinity Fcγ receptor locus (**Figure 3A** and **Supplementary Figure 3A**) are associated with diseases in which B cell depletion is a common therapeutic strategy (48), we hypothesized that B cell autoreactivity may contribute to pathology driven by impaired *TNFAIP3* function. Supporting this, antinuclear autoantibodies were previously described in A20-deficient mice (14) and in humans with A20 haploinsufficiency (30–32). Consistent with disease in mice with a CD19 Cre-driven *Tnfaip3* deficiency (16–18), transcriptomic profiling of B cells in A20^CD11c-ko^ mice indicated enhanced B cell receptor signaling and an upregulation of genes involved in antigen presentation (**Figure 3B**). These B cells also transcribed increased levels of *Ighg1* and *Igha* (**Supplementary Figure 3B**), in line with findings in A20^DNGR1-ko^ mice (55). Potentially amplifying these antibody responses, Fcγ receptor expression was markedly increased in myeloid cells at both transcriptional (**Supplementary Figures 3C, D**) and protein level (**Figure 3C**).

To evaluate the role of IgGs in systemic inflammation, we crossbred A20^CD11c-ko^ mice with mice lacking the neonatal Fc receptor (FcRn, encoded by *Fcgrt*, Fc gamma receptor and transporter) (39). Disrupted IgG recycling and trafficking by deficiency in FcRn resulted in significantly reduced levels of total, anti-dsDNA, and anti-cardiolipin IgGs in A20^CD11c-ko^ mice (**Figure 3D**). In contrast, IgA antibody concentrations remained unchanged. Confocal imaging on kidney revealed that FcRn deficiency diminished glomerular IgG deposition but did not affect deposition of IgA and IgM in comparison to FcRn-sufficient A20^CD11c-ko^ mice, while C3 deposition was modestly increased (**Figures 3E, F**). Histopathological evaluation of glomeruli from A20^CD11c-ko^ mice showed mesangial matrix expansion without overt glomerulonephritis, accompanied by large inflammatory infiltrates, and FcRn deficiency did not ameliorate this phenotype (**Figures 3G, H**).

**Figure 4:**
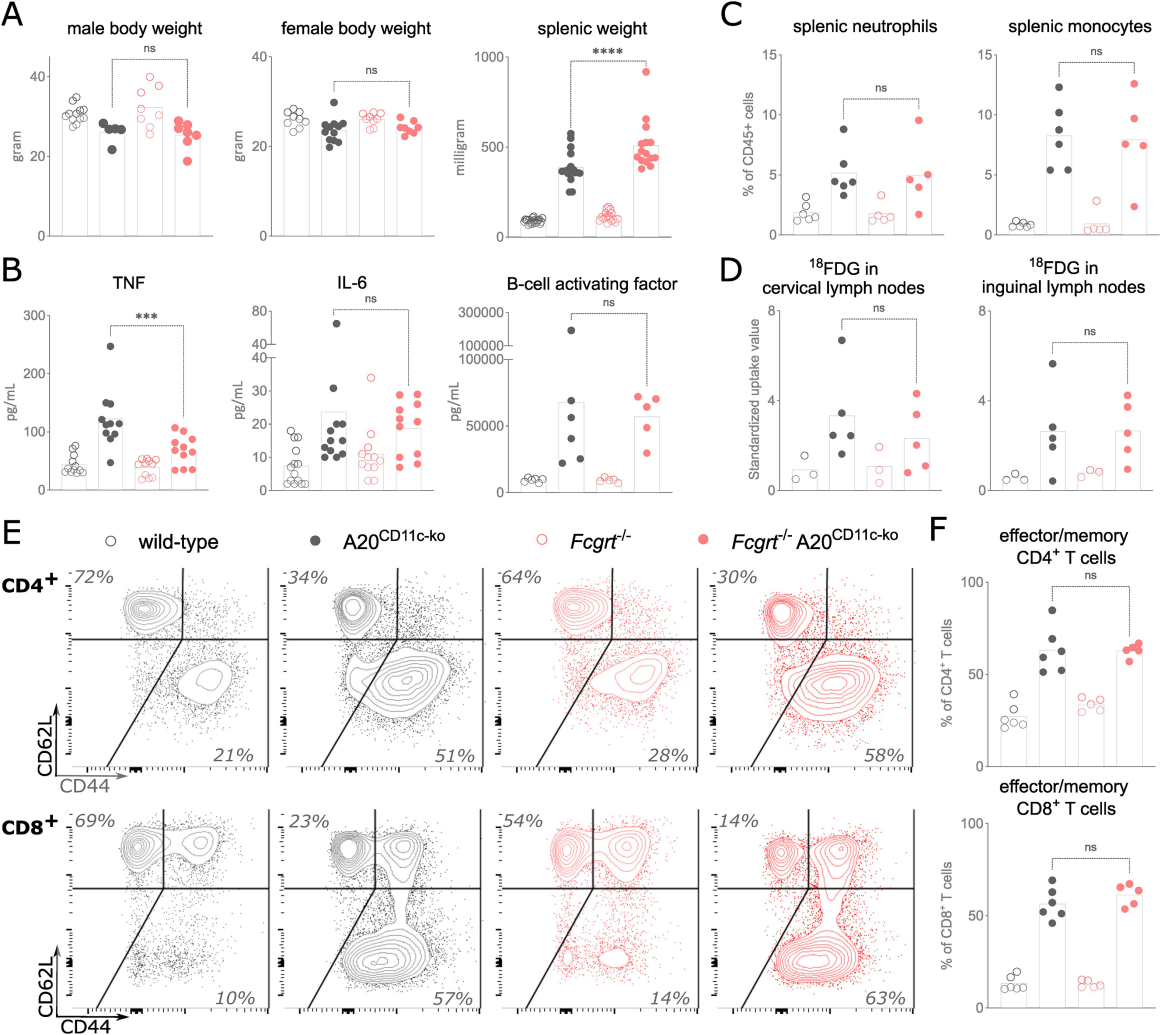
Systemic inflammation in A20^CD11c-ko^ mice occurs independently of IgG autoantibodies. **(A)** Body and spleen weights in 18–24 week old wild-type, A20^CD11c-ko^, *Fcgrt*^-/-^, and *Fcgrt*^-/-^ A20^CD11c-ko^ mice. **(B)** Serum cytokine levels by genotype. **(C)** Quantification of neutrophils and Ly6C^hi^ monocytes in spleen, expressed as percentage of CD45^+^ cells. **(D)** Fluorodeoxyglucose uptake in cervical or inguinal lymph nodes. **(E, F)** CD4^+^ and CD8^+^ T cell differentiation in spleen, with representative flow cytometry plots **(E)** and quantification of CD44^+^ CD62L^-^ T lymphocytes **(F)**. Statistical testing was carried out using two-way ANOVAs with Šídák’s multiple comparison testing. ns = non-significant; ***p < 0.001; ****p < 0.0001.

Next, we examined whether global inflammation in A20^CD11c-ko^ mice was improved by the reduction of autoreactive IgGs. Both FcRn-sufficient and FcRn-deficient A20^CD11c-ko^ mice exhibited cachexia and splenomegaly, with the latter even showing a slight increase in the absence of FcRn (**Figure 4A**). While serum TNF levels were modestly reduced, other pro-inflammatory cytokines, such as IL-6 and B-cell activating factor (BAFF or BLyS), remained elevated in FcRn^-/-^ A20^CD11c-ko^ mice (**Figure 4B**). No improvement was observed in myeloproliferation due to A20 deficiency (**Figure 4C**; gating see **Supplementary Figure 4**), and positron emission tomography (PET) scans indicated unaltered hypermetabolic lymphadenopathies (**Figure 4D**). Effector/memory CD4^+^ and CD8^+^ T cells were expanded in A20^CD11c-ko^ mice, and this phenotype was unchanged in FcRn-deficient A20^CD11c-ko^ mice (**Figures 4E, F**). Collectively, these data indicate that IgG autoantibodies, including anti-dsDNA IgGs, are not key drivers of systemic inflammation in the context of deficient *Tnfaip3* function.

### 3.4 A20-driven immune hyperactivation occurs independently of B cells

To assess the contribution of non-IgG antibodies, we generated B cell-deficient A20^CD11c-ko^ mice through crossbreeding with µMt mice, which have a B cell developmental arrest due to a premature stop codon in both IgM (µ) heavy chains (40). While some studies have reported the generation of antibodies in the absence of IgM (56, 57), we could not detect B cells in the spleen or CD138^+^ plasma cells in the bone marrow of µMt or µMt A20^CD11c-ko^ mice (**Supplementary Figure 5A**). These mice also lacked antibodies in circulation and did not exhibit any glomerular immune complex or complement deposition (**Supplementary Figures 5B–F**).

**Figure 5:**
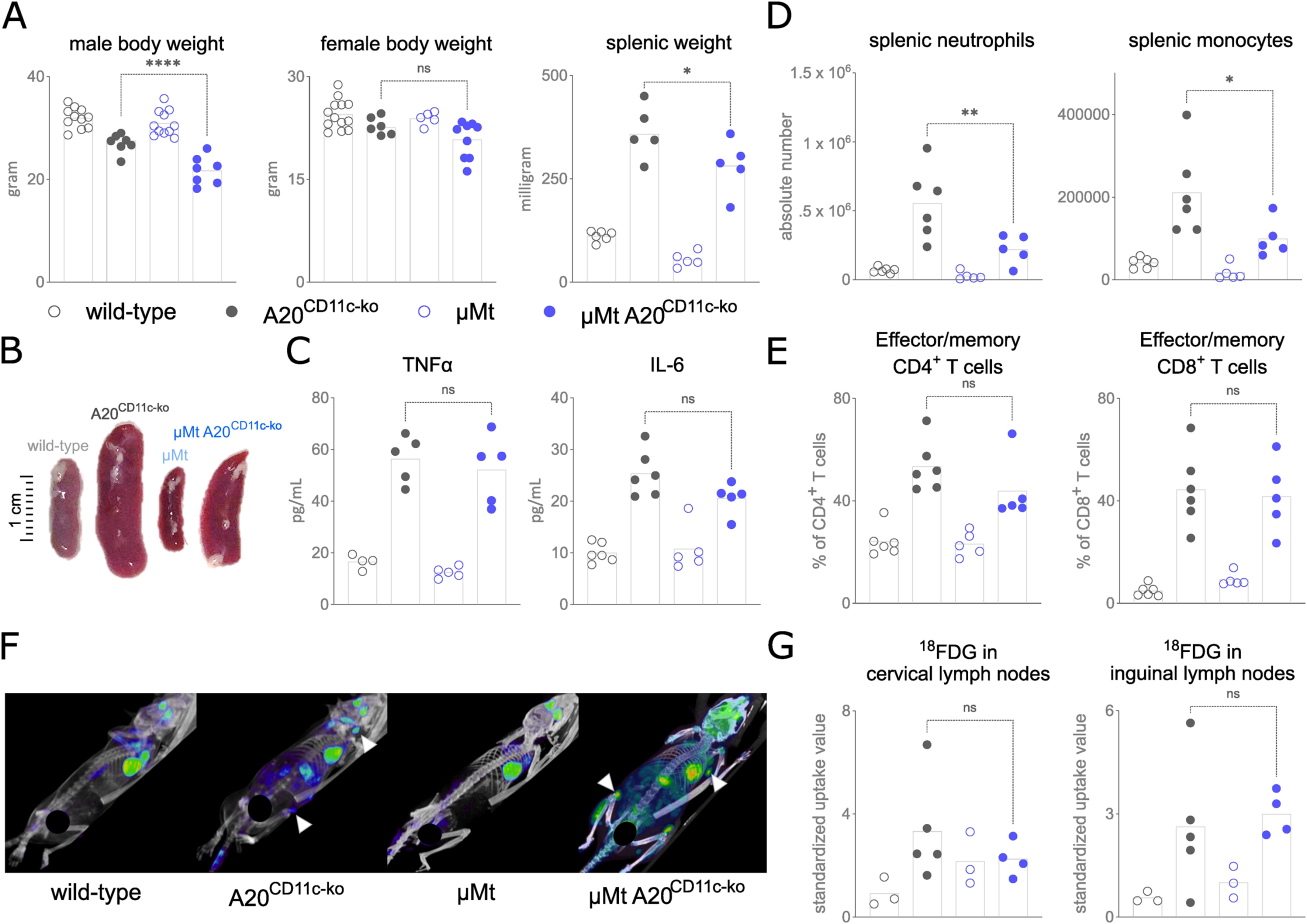
Antibody and B cell deficiency does not salvage systemic inflammation in mice with CD11c-driven A20-deficiency. **(A)** Body and splenic weights across wild-type, A20^CD11c-ko^, µMt, and µMt A20^CD11c-ko^ mice. **(B)** Representative image of spleens per genotype. **(C)** Circulating cytokines quantified by enzyme-linked immunosorbent assays. **(D)** Absolute numbers of neutrophils and Ly6C^hi^ monocytes in spleen. **(E)** Differentiation of CD4^+^ and CD8^+^ T cells to CD62L^-^ CD44^+^ phenotype. **(F, G)** Uptake of fluorodeoxyglucose (^18^FDG) assessed on PET-CT scanning, with white triangles marking lymphadenopathies **(F)** in which tracer uptake was quantified **(G)**. Two-way ANOVAs corrected using Šídák’s multiple comparison were used to assess significance. ns = non-significant; *p < 0.05; **p < 0.01; ****p < 0.0001.

However, the absence of B cells did not ameliorate weight loss in A20^CD11c-ko^ mice, which was slightly worse in B cell-deficient A20^CD11c-ko^ males (**Figure 5A**). Splenomegaly did improve slightly, likely resulting from the loss of B cells which make up a large number of CD45^+^ cells in the spleen (**Figures 5A, B**). Loss of antibodies did not rescue the elevated levels of circulating cytokines observed in A20^CD11c-ko^ mice (**Figure 5C**), and the expansion of myeloid cells in the spleen remained much higher in A20-deficient µMt mice compared to A20-sufficient µMt mice (**Figure 5D**). While B cells were suggested to play a role in antigen presentation in our single-cell experiment (**Supplementary Figure 3A**), we found that T cell differentiation to an effector phenotype did not improve either (**Figure 5E**). Reflecting the ongoing hyperinflammation, PET-CT imaging underscored persistent hypermetabolism in cervical and inguinal lymph nodes in A20^CD11c-ko^ mice, regardless of B cell presence (**Figures 5F, G**).

### 3.5 A20-driven systemic inflammation arises independently of adaptive immunity

**Figure 6:**
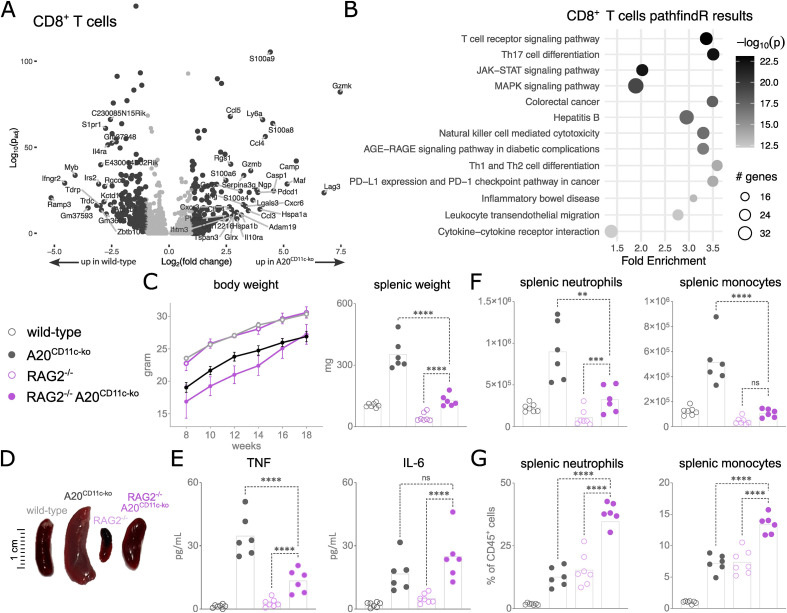
A20-dependent immune hyperactivation arises independently of B and T cells. **(A)** Differentially expressed genes in CD8^+^ T cells from wild-type or A20^CD11c-ko^ mice. **(B)** Enrichment analysis on splenic CD8^+^ T cells from A20^CD11c-ko^ versus wild-type mice. **(C)** Body and splenic weights across genotypes (body weight measurements: wild-type, n = 38; A20^CD11c-ko^, n = 27; RAG2^-/-^, n = 4; RAG2^-/-^ A20^CD11c-ko^, n = 9). **(D)** Representative image of spleens per genotype. **(E)** Circulating cytokines quantified by enzyme-linked immunosorbent assays. **(F, G)** Neutrophils and Ly6C^hi^ monocytes in spleen, quantified as the absolute number **(F)** or percentage of CD45^+^ cells **(G)**. Two-way ANOVAs corrected using Šídák’s multiple comparison were used to assess significance. ns = non-significant; **p< 0.01; ****p< 0.0001.

At the single-cell level, CD8^+^ T cells from A20^CD11c–ko^ mice displayed the most pronounced transcriptional shift among splenic immune populations. Differential expression and pathway analyses revealed increased cytotoxic activity (*Gzmb*, *Gzmk*, *Ifng*), enhanced chemokine programs (upregulation of *Ccl3/4/5*, *Cxcr6* and downregulation of *S1pr1*), and features of stimulation and exhaustion (*Pdcd1*, *Lag3*, *Maf*), in A20^CD11c-ko^ CD8^+^ T cells (**Figures 6A, B**, **Supplementary Figure S6A**). CD4^+^ T cells displayed similar TNF- and JAK–STAT-driven activation patterns, though the transcriptional changes were less pronounced than in CD8^+^ T cells (**Supplementary Figure 6B**).

Given the strong effector skewing of T cells, which are partially targeted by the CD11c-Cre, and the persistence of this phenotype even in the absence of B cells (**Figure 5E**), we investigated whether T cells are required for the inflammatory syndrome triggered by loss of A20. We therefore generated A20^CD11c-ko^ mice lacking *RAG2*, which is required for V(D)J recombination and T and B cell development (41).

The absence of adaptive lymphocytes did not prevent cachexia in A20^CD11c-ko^ mice, and splenomegaly remained pronounced in RAG2^-/-^ animals lacking A20 (**Figures 6C, D**). Serum TNF levels were markedly reduced in RAG2^-/-^ A20^CD11c–ko^ mice but remained substantially elevated relative to RAG2^-/-^ controls (**Figure 6E**). IL-6 concentrations were unaffected by the presence or absence of adaptive immunity. Interestingly, pathway analysis of the CITE-seq dataset did not reveal consistent enrichment of TNF- or IL-6-responsive transcriptional programs across splenic immune populations (**Supplementary Figure 6C**), suggesting that these cytokines may not constitute the dominant inflammatory pathways downstream of A20 deficiency. In contrast, transcriptional signatures associated with type I and type II interferon signaling, as well as NLRP3 inflammasome activation, were broadly enriched across multiple immune cell populations (**Supplementary Figure 6D**), implicating these pathways as potential contributors to disease pathogenesis. In the spleen, the absolute expansion of neutrophils and monocytes driven by A20 deficiency was strongly blunted in RAG2^-/-^ mice, although their relative frequencies remained clearly increased (**Figures 6F, G**). These findings show that A20-dependent immune hyperactivation arises independently of antigen-receptor–expressing lymphocytes.

### 3.6 Microbial signals promote IgA class switching but are dispensable for systemic A20-driven inflammation

**Figure 7:**
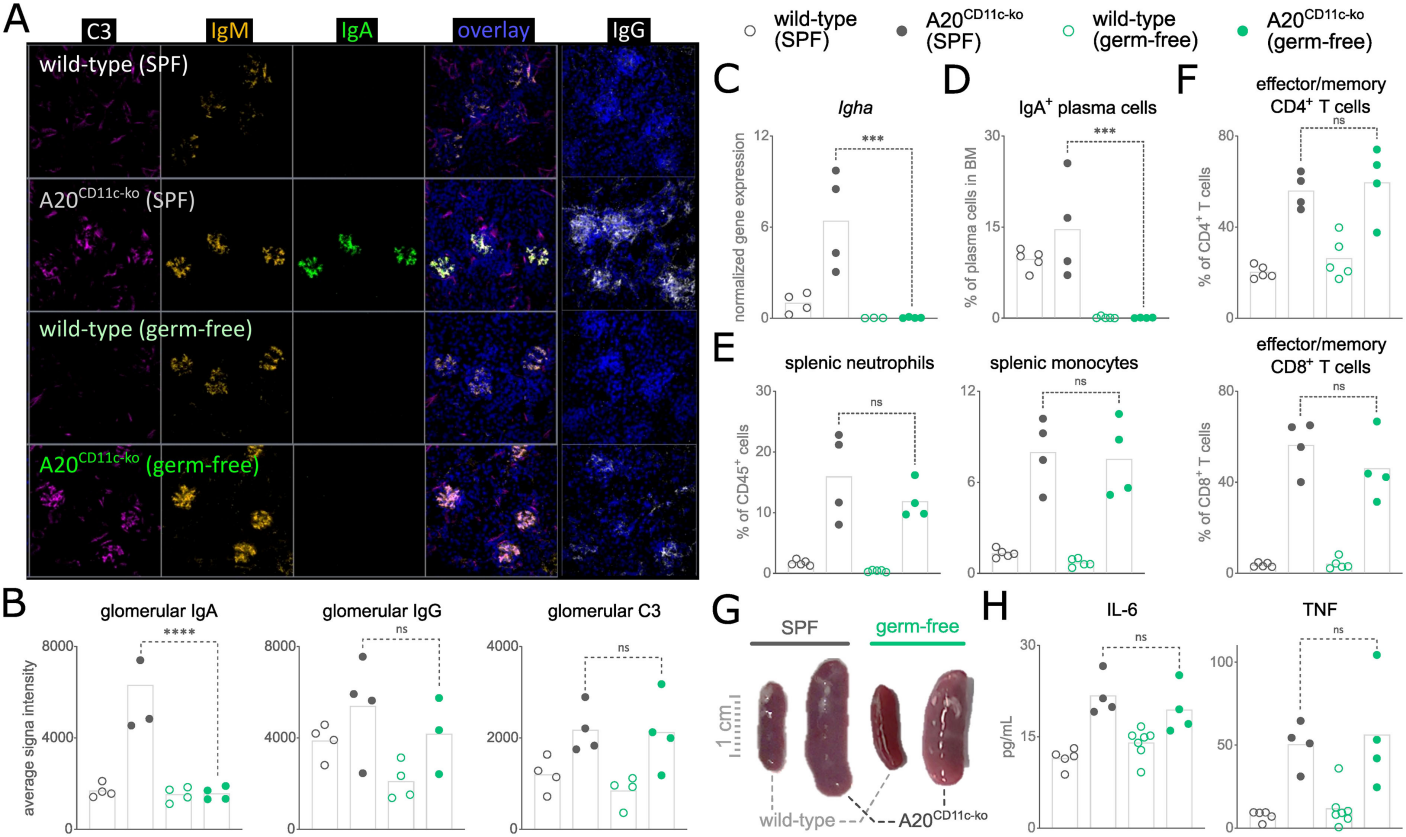
Microbial triggers drive IgA class switching but are redundant for systemic inflammation. **(A, B)** Confocal images of glomerular deposition of immunoglobulin and complement **(A)**, with quantification of fluorescent signal intensity for each genotype and microbial status **(B)**. **(C)** Quantitative PCR analysis of IgA heavy chain (*Igha*) expression in the small intestine. **(D)** Proportion of IgA^+^ plasma cells in the bone marrow, determined by flow cytometry and expressed as a percentage of total CD138^+^ plasma cells. **(E)** Flow cytometric quantification of neutrophils and Ly6C^hi^ monocytes as a fraction of splenic immune cells. **(F)** Effector/memory phenotype of splenic CD4^+^ (top) or CD8+ (bottom) T cells analysed by flow cytometry. **(G)** Representative spleen images from each genotype under specific-pathogen-free (SPF) or germ-free conditions. **(H)** Serum cytokine concentrations across genotypes and microbial environments. Statistical evaluation was performed using two-way ANOVAs with Šídák’s multiple comparison. ns = non-significant; ***p < 0.001; ****p < 0.0001.

Given the strongly elevated titers of autoreactive and total IgA, as well as a divergent phenotype of A20^CD11c-ko^ mice observed in different animal facilities (14, 15), we next focused on the microbiome as a potential trigger of systemic inflammation. We rederived A20^CD11c-ko^ mice to axenic (completely germ-free) conditions and compared these to wild-type and A20^CD11c-ko^ mice in specific-pathogen-free (SPF) conditions. An absence of microbial colonization resulted in significantly reduced IgA deposition in the kidneys and an absence of circulating IgA, while IgG autoreactivity and glomerular complement deposition were unaltered in germ-free A20^CD11c-ko^ mice (**Figures 7A, B**, **Supplementary Figure S7A**). We found that reduced IgA levels in germ-free mice resulted from defective intestinal as well as defective systemic IgA production (**Figures 7C, D**). The absence of IgA responses correlated with decreased expression of antimicrobial peptides (*Reg3g*) and *Il17a* in the gut (**Supplementary Figure 7B**), which was previously shown to regulate intestinal antibody and systemic IgA responses (58–60).

Organ-wide inflammation, however, was not alleviated under germ-free conditions. The number of intestinal neutrophils was decreased in germ-free compared to SPF A20^CD11c-ko^ mice, but lamina propria monocytes as well as splenic myeloid cells remained strongly increased in A20-deficient mice regardless of colonization status (**Figure 7E**, **Supplementary Figure S7C**). Spontaneous skewing of CD4^+^ and CD8^+^ to an effector/memory phenotype, as observed in SPF A20^CD11c-ko^ mice, did not depend on microbial stimuli either (**Figure 7F**, **Supplementary Figure S7D**). Finally, splenic weight and cytokines in circulation remained strongly elevated in A20^CD11c-ko^ mice (**Figures 7G, H**), indicating that systemic inflammation depends on sterile rather than exogenous triggers.

## 4 Discussion

*TNFAIP3*/A20 regulates inflammation in an evolutionary conserved manner, underscoring its essential role in immune homeostasis. To elucidate global mechanisms underlying *TNFAIP3*-associated conditions, we utilized mice deficient in A20 in CD11c-expressing cells. Unexpectedly, this genetic approach reduced *Tnfaip3* expression across all major immune populations, extending beyond dendritic cells. Consequently, the phenotype observed in A20^CD11c-ko^ mice is unlikely to solely reflect dendritic cell-intrinsic effects, but rather a combination of cell-intrinsic and cell-extrinsic consequences of broad immune A20 insufficiency. In this regard, the model may more closely resemble the widespread reduction in A20 expression observed in patients with A20 haploinsufficiency than a selective dendritic cell-specific deletion.

Given the therapeutic potential of FcRn inhibition in IgG-mediated diseases (61), we abolished FcRn-mediated IgG recycling in A20^CD11c-ko^ mice. As anticipated, this reduced the levels of IgG (auto)antibodies and their glomerular deposition. However, organ-wide inflammation remained unchanged in the absence of autoreactive IgGs. Supporting these findings, systemic immune dysregulation was unaffected even in the complete absence of B cells or combined B and T cell deficiency, indicating that disease driven by broad A20 deficiency does not require adaptive immunity nor microbial triggers.

The contribution of individual immune cell populations to disease pathogenesis in A20^CD11c-ko^ mice remains difficult to disentangle. More specific targeting of dendritic cells, using a *Clec9a* (DNGR1)-Cre, induces spontaneous T- and B-cell activation (55, 62), demonstrating that *Tnfaip3* in dendritic cells is necessary to restrain adaptive immune dysregulation. However, severe manifestations observed in A20^CD11c-ko^ mice such as weight loss, hematological abnormalities, and immune complex deposition have not been reported in these models. Conversely, myeloid-restricted deletion using *Lyz2* (LysM)-Cre results in inflammatory arthritis that develops independently of adaptive immunity (11). Interestingly, although mice with A20-deficient myeloid cells do not develop weight loss, they are protected from high-fat diet-induced obesity and insulin resistance (63). These observations suggest that the phenotype of A20^CD11c-ko^ mice arises from the combined effects of A20 deficiency across multiple immune compartments rather than from disruption of a single lineage.

The redundancy of autoantibodies in immune dysregulation in A20^CD11c-ko^ mice was surprising, given that in humans, *TNFAIP3* polymorphisms predispose to multiple diseases usually considered as B cell-driven, including SLE, rheumatoid arthritis, and systemic sclerosis. Moreover, *TNFAIP3* mutations or epigenetic alterations leading to sustained NF-κB signaling are known to promote B cell survival and lymphoma development (64), and CD19-Cre driven deletion of A20 mediates autoantibody formation and prevents B cell apoptosis (16). Given that B cells are abundantly targeted by CD11c-driven Cre recombinase, a similar mechanism could be reflected in A20^CD11c-ko^ mice. Nonetheless, our data indicate that IgG autoantibodies and B cells are dispensable for the development of organ-wide inflammation in this model. This might be due to insufficient pathogenic IgG antibody titers, or because most autoantibodies were of the IgG1 isotype, which preferentially binds the inhibitory rather than activating Fcγ receptors in mice (65, 66).

Environmental factors, including infections, smoking, and the microbiome, play central roles in immune-mediated diseases (33, 34, 67, 68). Given the profound IgA bias in A20^CD11c-ko^ mice, which is also the dominant antibody isotype in *TNFAIP3*-linked disorders such as SLE, IBD, and Behçet’s disease (69), and the observation of distinct disease manifestations across different animal facilities (14, 15), we hypothesized that commensal microbes could modulate autoimmune features and contribute to disease heterogeneity. However, while germ-free rederivation alleviated IgA skewing, it did not alter overall disease in A20^CD11c-ko^ mice. These findings align with studies in mice lacking *Tnfaip3* in LysM-expressing cells and in A20 zinc finger 7 mutant mice, in which microbial triggers were similarly dispensable for arthritis and dactylitis development (7, 50). It is important to note that IgA effector functions differ largely between species, as FcαR, the activating IgA receptor, is not encoded by the mouse genome (70, 71). Thus, while microbiome-dependent autoreactive IgA might not elicit strong effector functions in mice, it might still stimulate FcαR^+^ neutrophils, eosinophils, and monocytes in human patients (72). Thus, our data do not exclude the possibility that IgA contributes to pathology in patients with inadequate A20 activity. In humans, IgA may act as a biomarker of immune activation, a tissue-specific effector molecule, or a modifier of ongoing systemic inflammation through FcαRI-dependent mechanisms.

The redundancy of autoantibodies and commensals indicates that alternative pathways drive inflammation in A20^CD11c-ko^ mice. The clinical benefit of colchicine in A20 haploinsufficiency patients hints at a role for neutrophils in disease pathogenesis. However, we previously demonstrated that *Padi4*-dependent neutrophil extracellular traps (NETs) do not contribute to disease features in either A20^CD11c-ko^ or A20^LysM-ko^ mice (6). Other therapeutic strategies have targeted cytokine pathways. TNF blockade has been applied in patients with HA20 (28) and shown efficacy in colitis models with conditional A20 deficiency (37, 73). Notably, despite a marked reduction in circulating TNF levels in RAG2^-/-^ A20^CD11c-ko^ mice, systemic inflammation remained largely unchanged. Combined with the lack of enrichment of TNF-responsive transcriptional signatures across splenic immune populations, these findings argue against TNF as the principal driver of disease in this model. Similar observations have been made in A20^LysM-ko^ mice, where TNF receptor deficiency did not protect against arthritis development (11).

Janus kinase (JAK) inhibitors, which interfere with the signaling of multiple cytokines, have shown efficacy in patients with increased type 1 interferon signatures (74, 75). Beyond this, excessive interferon-γ (IFNγ) has emerged as a key driver in several *Tnfaip3*-deficient models (13, 20, 76, 77). Consistent with these observations, pathway analysis of splenocytes from A20^CD11c-ko^ mice revealed broad enrichment of both type I and type II interferon-responsive transcriptional programs across multiple immune cell populations. Of note, the *TNFAIP3* locus lies in close proximity to *IFNGR1* on chromosome 6, and their co-regulation may simultaneously decrease *TNFAIP3* expression while increasing the sensitivity for IFNγ in certain patients (78, 79). In addition, evidence from both experimental models (9, 12) and patients with A20 haploinsufficiency, who frequently display elevated circulating IL-1 and IL-18 levels (29, 31), has implicated NLRP3 inflammasome activation in disease pathogenesis. Supporting this, our pathway analyses identified enrichment of NLRP3 inflammasome-associated transcriptional signatures. Together, these data highlight interferon signaling and pyroptosis as potential therapeutic targets in A20^CD11c-ko^ mice, warranting additional investigation.

In contrast, despite the success of deep B celldepleting therapies in the treatment of refractory autoimmune diseases such as rheumatoid arthritis, SLE and systemic sclerosis (80–82), our findings indicate that B cells and autoantibodies are dispensable for disease development in A20^CD11c-ko^ mice. These observations suggest that therapeutic strategies targeting adaptive immunity may not address the core inflammatory pathways underlying disease pathogenesis, they could still prove beneficial for specific immune complex-mediated manifestations, such as glomerulonephritis or arthritis.

Overall, our data support the notion that autoantibodies are more likely to represent biomarkers than primary drivers of pathology in the context of *TNFAIP3* deficiency. Furthermore, neither adaptive immunity nor the microbiota proved essential for the development of systemic inflammation in A20^CD11c-ko^ mice. Together, these findings indicate that inflammatory pathways operating independently of antigen receptor-expressing lymphocytes and microbial stimuli are sufficient to drive pathology in the context of impaired A20 function. While caution is warranted when extrapolating these observations to patients, our results support the concept that immune dysregulation associated with TNFAIP3 deficiency may be more appropriately classified as autoinflammatory in nature.

## Data Availability

The raw sequencing data and processed object generated in this publication are accessible through GEO under accession number GSE338131. The scripts used for analyzing the CITE-sequencing data of wild-type or A20^CD11c-ko^ splenocytes can be found on https://doi.org/10.5281/zenodo.21258612. The mouse spleen CITE-seq dataset can be accessed via our online tool: www.single-cell.be/data-explorer/browser/861b2647-304e-4280-ae67-e27772dbcf63. Additional data can be found in the **Supplementary Material**.
