# Supplementary material for: TNFAIP3/A20 dysfunction drives innate and sterile hyperinflammation
Source: Front Immunol. 2026 Jul 17;17:1856810. doi: 10.3389/fimmu.2026.1856810 (PMC13423863; doi:10.3389/fimmu.2026.1856810)
Supplement: Supplementary file 1 [file SupplementaryFile1.zip › Suppl Data 5 Antibody List.pdf]

**Antigen (species raised, clone), conjugation**	**Brand**	**Catalog number**
Anti-mouse BST2 (rat, 120-G8), AF647	*Produced in house*	*Not applicable*
Anti-mouse BST2 (rat, 120-G8), FITC	*Produced in house*	*Not applicable*
Anti-mouse C3 (rat, 11H9), APC	Novus Biologicals	NB200
Anti-mouse CD11b (rat, M1/70), BUV395	BD Biosciences	563553
Anti-mouse CD11b (rat, M1/70), FITC	Invitrogen	11-0112-82
Anti-mouse CD11c (Armenian hamster, HL3), FITC	BD Biosciences	553801
Anti-mouse CD11c (Armenian hamster, N418), BV605	BioLegend	117334
Anti-mouse CD11c (Armenian hamster, N418), PE-Cy7	BioLegend	117318
Anti-mouse CD11c (Armenian hamster, N418), PE-eFluor610	eBioscience	61-0114-82
Anti-mouse CD138 (Rat, 281-2), BV711	BioLegend	142519
Anti-mouse CD172a (rat, P84), PE-Cy7	BioLegend	144008
Anti-mouse CD16 (rat, 275003), BV786	Bio-Teche	FAB19601A
Anti-mouse CD16.2 (Armenian Hamster, 9E9), APC	BioLegend	149535
Anti-mouse CD19 (Rat, 1D3), AF700	Invitrogen	56-0193-82
Anti-mouse CD19 (Rat, 1D3), APC	BD Biosciences	550992
Anti-mouse CD19 (rat, 1D3), BV786	BD Biosciences	563333
Anti-mouse CD19 (Rat, 1D3), FITC	Avantor	35-0193
Anti-mouse CD19 (rat, 1D3), PE-Cy5	eBioscience	15-0193-83
Anti-mouse CD26 (rat, H194-112), BUV737	BD Biosciences	741729
Anti-mouse CD26 (rat, H194-112), FITC	Biolegend	137806
Anti-mouse CD3e (rat, 17A2), AF700	BioLegend	100216
Anti-mouse CD3e (Armenian hamster, 145-2C11), BUV737	BD Biosciences	612771
Anti-mouse CD3e (Armenian hamster, 145-2C11), FITC	BioLegend	100306
Anti-mouse CD3e (Armenian hamster, 145-2C11), PE	eBioscience	12-0031-82
Anti-mouse CD3e (Armenian hamster, 145-2C11), PE-Cy5	eBioscience	15-0031-83
Anti-mouse CD3e (Armenian hamster, 145-2C11), PE-Cy7	BioLegend	100320
Anti-mouse CD32b (mouse, AT130-2), PE	Invitrogen	12-0321-82
Anti-mouse CD4 (Rat, GK1.5), BUV395	BD Biosciences	563790
Anti-mouse CD4 (Rat, GK1.5), PE-eFluor610	Invitrogen	61-0042-82
Anti-mouse CD43 (rat, S7), BUV737	BD Biosciences	612840
Anti-mouse CD44 (rat, IM7), BV650	BioLegend	103049
Anti-mouse CD44 (rat, IM7), redFluor710	Avantor	TONB80-0441
Anti-mouse CD45 (rat, 30-F11), BV510	BioLegend	103138
Anti-mouse CD45 (rat, 30-F11), PE-eFluor610	Invitrogen	61-0451-82
Anti-mouse CD45R (rat , RA3-6B2), BV605	BD Biosciences	563708
Anti-mouse CD62L (rat, MEL-14), BV421	eBioscience	11-0621-85
Anti-mouse CD62L (rat, MEL-14), PE	BioLegend	104408
Anti-mouse CD64 (mouse, X54-5/7.1), BV711	BioLegend	139311
Anti-mouse CD88 (rat, 20/70), PE	BioLegend	135806
Anti-mouse CD88 (rat, 20/70), PerCP-Cy5.5	BioLegend	135813
Anti-mouse CD8a (rat, 53-6.7), PE-Cy7	BioLegend	100722
Anti-mouse CD8a (rat, 53-6.7), PerCP-Cy5.5	eBioscience	45-0081-82
Anti-mouse CD90 (rat, 30-H12), BUV395	BD Biosciences	740205
Anti-mouse CD95 (hamster, Jo2), PE-Cy7 conjugated	BD Biosciences	557653
Anti-mouse F4/80 (rat, BM8), biotin	Invitrogen	13-4801-85
Anti-mouse F4/80 (rat, BM8), BV786	BioLegend	123141

Anti-mouse GL7 (rat, GL7), AF647	BioLegend	144606
Anti-mouse I-A/I-E (rat, M5/114.15.2), FITC	Invitrogen	11-5321-85
Anti-mouse I-A/I-E (rat, M5/114.15.2), APC	BioLegend	107614
Anti-mouse I-A/I-E (rat, M5/114.15.2), PerCP-Cy5.5	BioLegend	107626
Anti-mouse IgA (goat, polyclonal), DyLight488	Abcam	ab97011
Anti-mouse IgA (rat, C10-3), FITC	BD Biosciences	559354
Anti-mouse IgD (rat, 11-26c.2a), BD Horizon V450	BD Biosciences	560869
Anti-mouse IgG (donkey, polyclonal), Cy5	Jackson ImmunoResearch	715-175-150
Anti-mouse IgM (goat, polyclonal), Cy3	Jackson ImmunoResearch	115-166-075
Anti-mouse Ly6C (rat, AL21), PE	BD Biosciences	560592
Anti-mouse Ly6C (rat, HK1.4), eFluor450	eBioscience	48-5932-82
Anti-mouse Ly6G (rat, 1A8), AF700	BioLegend	127622
Anti-mouse Ly6G (rat, 1A8), BUV563	BD Biosciences	612921
Anti-mouse Ly6G (rat, 1A8), BV650	BD Biosciences	740554
Anti-mouse Ly6G (rat, 1A8), FITC	BD Biosciences	551460
Anti-mouse NK1.1 (mouse, PK136), PE-Cy5	BioLegend	108716
Anti-mouse Siglec F (rat, E50-2440), BUV395	BD Biosciences	740280
Anti-mouse Siglec H (rat, eBio440c), eFluor660	Invitrogen	50-0333-82
Anti-mouse XCR1 (mouse, ZET), BV650	BioLegend	148220
Anti-mouse γδTCR (Armenian hamster, GL3), FITC	BD Biosciences	553177
Fixable Viability Dye, eFluor780	eBioscience	65-0865-14
Streptavidin, BV605	BD Biosciences	563260
