# Supplementary material for: TNFAIP3/A20 dysfunction drives innate and sterile hyperinflammation
Source: Front Immunol. 2026 Jul 17;17:1856810. doi: 10.3389/fimmu.2026.1856810 (PMC13423863; doi:10.3389/fimmu.2026.1856810)
Supplement: Supplementary file 1 [file SupplementaryFile1.zip › Suppl Figures 1 - 7.pdf]

TNFAIP3/A20 dysfunction drives innate and sterile hyperinflammation

Karel F.A. Van Damme[1,2,3*], Pieter Hertens[4,5], Dorine Sichien[1,2,6], Katrien Van der Borght[1,2,7], Justine Van Moorleghem[1,2], Sofie De Prijck[1,2], Alex Klarenbeek[1,2], Els Louagie[6], Inés Lammens[1,2,8], Stijn Vanhee[1,2,8], Christian Vanhove[9], Pieter De Bleser[5,10], Steven Van Laecke[11], Amélie Dendooven[12], Hamida Hammad[1,2], Lars Vereecke[2,13,14], Dirk Elewaut[2,3,15], Geert van Loo[4,5,14,16], Bart N. Lambrecht[1,2,17,18*]

[1]Laboratory of Mucosal Immunology, VIB-UGent Center for Inflammation Research, Belgium
[2]Department of Internal Medicine and Pediatrics, Faculty of Medicine and Health Sciences, Ghent University, Belgium
[3]Department of Rheumatology, Ghent University Hospital, Ghent University, Belgium.
[4]Laboratory of Cellular and Molecular (Patho)physiology, VIB-UGent Center for Inflammation Research, Belgium
[5]Department of Biomedical Molecular Biology, Ghent University, Belgium.
[6]argenx, Belgium
[7]VIB Flow Core, VIB Center for Inflammation Research, Belgium
[8]Upper Airways Research Laboratory, Department of Head and Skin, Ghent University, Belgium
[9]IBiTech—Medisip—Infinity lab, Ghent University, Ghent, Belgium
[10]Data Mining and Modeling for Biomedicine Group, VIB-UGent Center for Inflammation Research, Belgium
[11]Renal Division, Department of Internal Medicine, Ghent University Hospital, Ghent, Belgium
[12]Division of Pathology, University Hospital Ghent, Belgium
[13]Host-Microbiota-Interaction Laboratory, VIB-UGent Center for Inflammation Research, Belgium10
[14]Cancer Research Institute Ghent, Ghent University, Belgium
[15]Molecular Immunology and Inflammation Laboratory, VIB-UGent Center for Inflammation Research, Belgium
[16]Ghent Gut Inflammation Group, Ghent University, Belgium
[17]Department of Pulmonary Medicine, Erasmus MC, The Netherlands
[18]Department of Respiratory Medicine, Ghent University Hospital, Belgium

* **Correspondence**:
Karel Van Damme, karel.vandamme@ugent.be; Bart Lambrecht, bart.lambrecht@ugent.be

Supplementary Data

Suppl. Data 1: Transcription factors with regulatory potential on *TNFAIP3* in humans and mice.

Suppl. Data 2: Cytokines modulating *Tnfaip3* expression in the Immune Dictionary.

Suppl. Data 3: Top differentially expressed proteins for populations in spleen.

Suppl. Data 4: Top differentially expressed genes for populations in spleen.

Suppl. Data 5: Antibody list.

Supplementary Figures

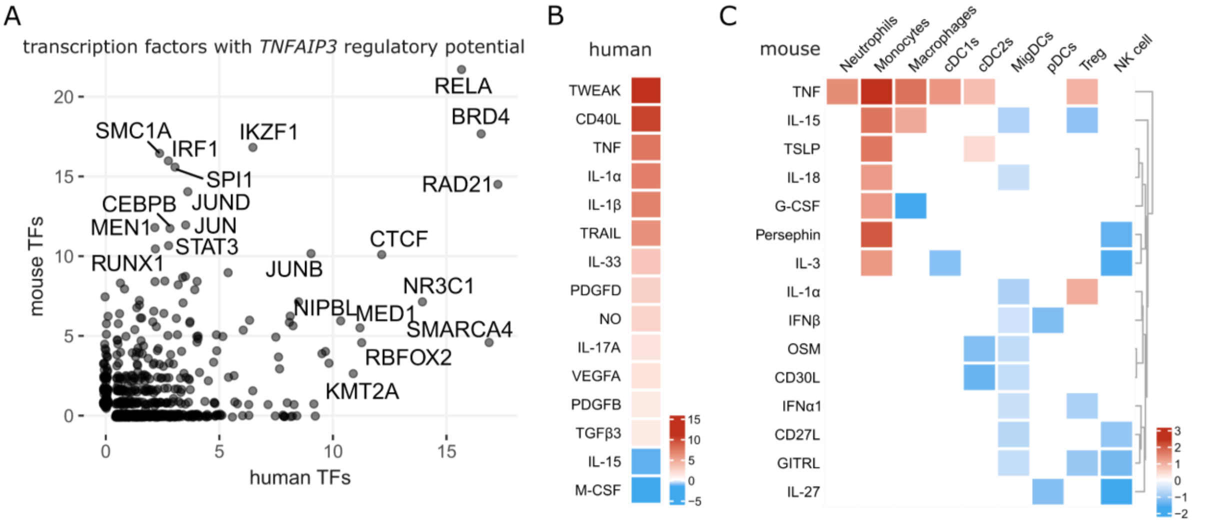

Figure S1: Comparative analysis of *TNFAIP3* regulation in humans and mice. (**A**) Transcription factors with regulatory potential on *TNFAIP3* based on compiled chromatin immunoprecipitation sequencing data. (**B-C**) Cytokines which modulate *TNFAIP3* expression according to CytoSig (B) or the Immune Dictionary (C).

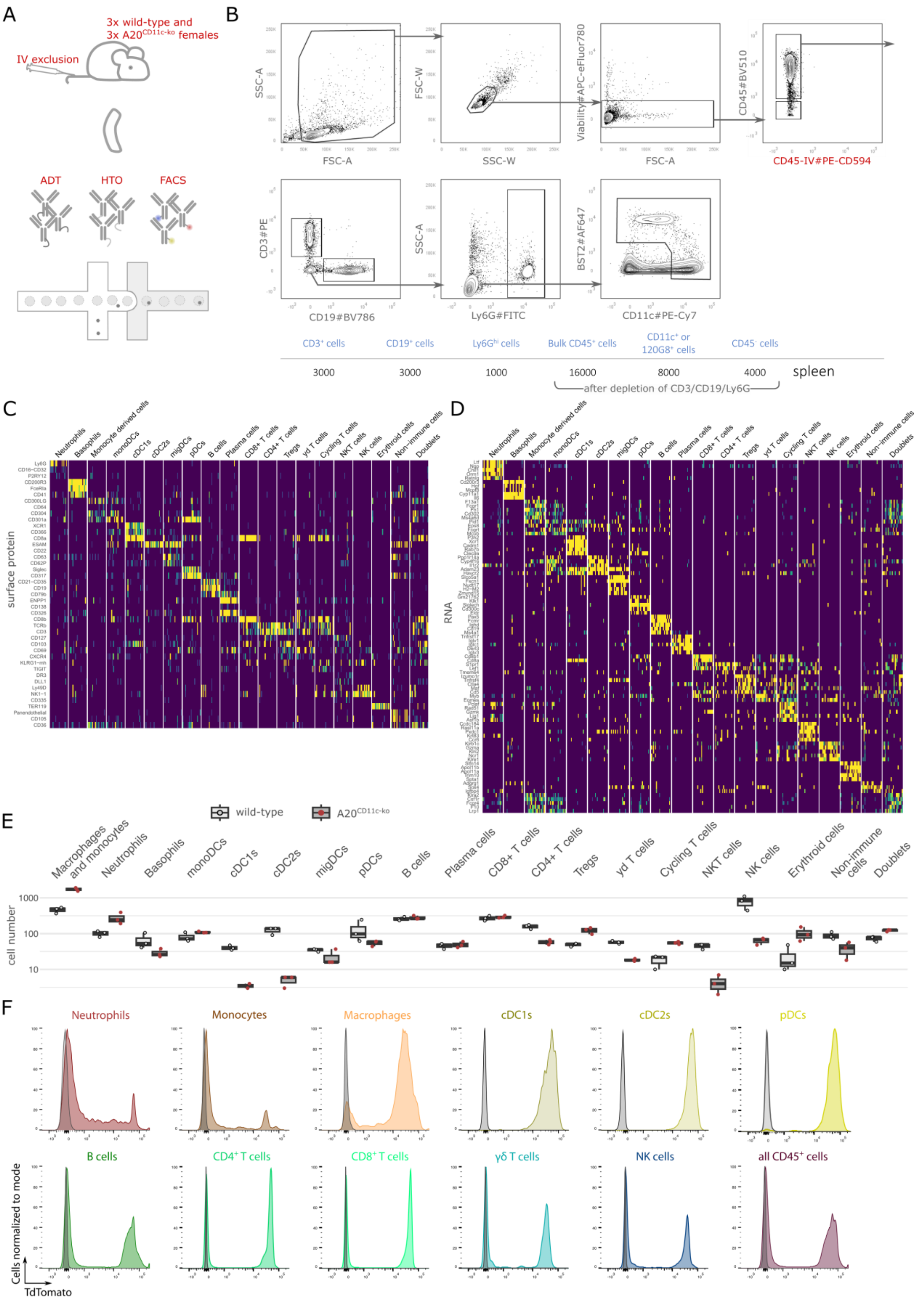

Figure S2: Systemic and multiomics evaluation of A20$^{CD11c\text{-}ko}$ mice. (**A**) Experimental set-up. (**B**) Gating strategy used during fluorescence-activated cell sorting (FACS). (**C-D**) Marker surface proteins (C) or genes (D) for cell populations identified in spleen. (**E**) Number of cells

across all populations in spleen, shown for each replicate. (**F**) TdTomato expression in CD11c-Cre reporter mice across splenic cell populations as measured by flow cytometry.

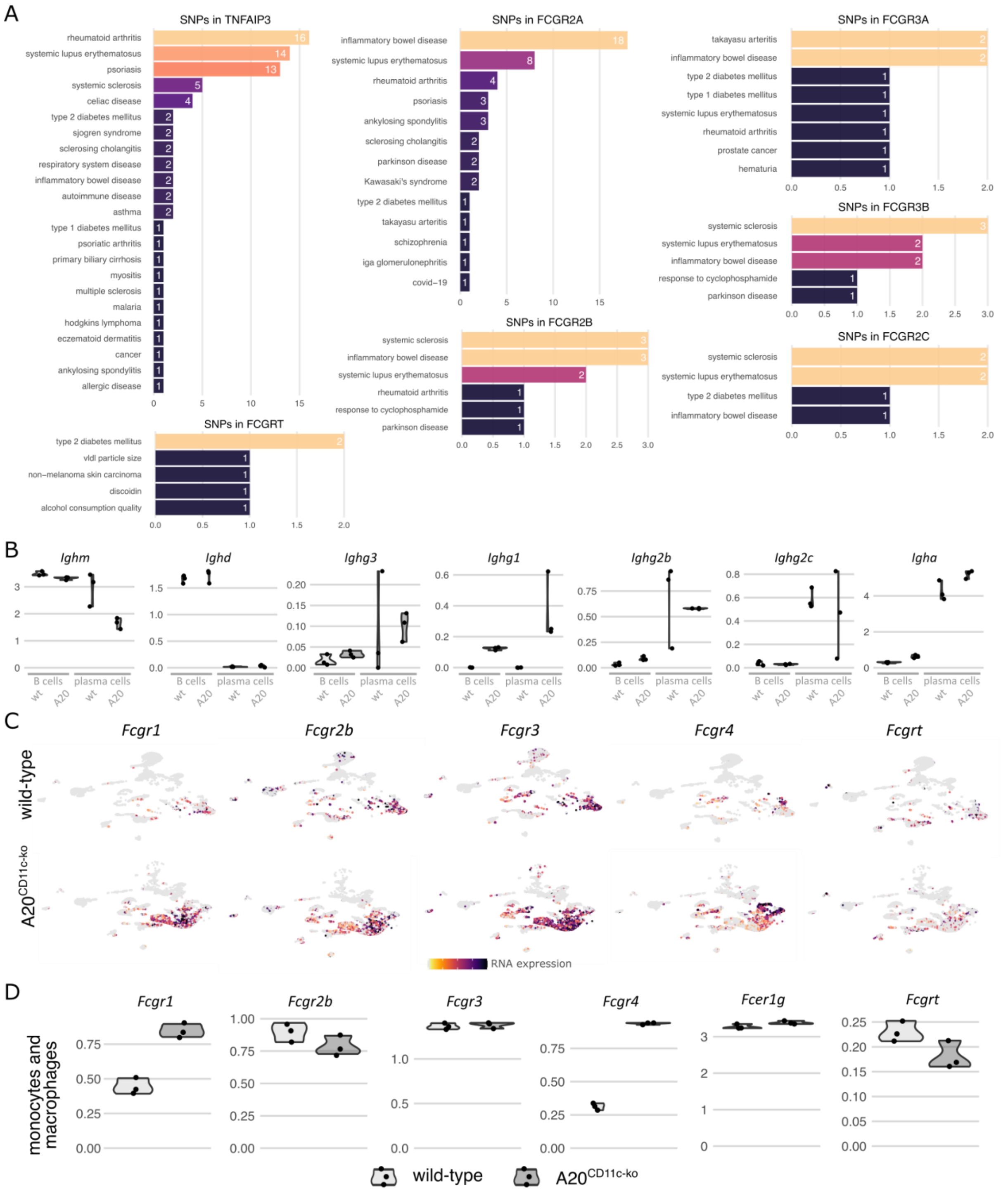

Figure S3: Antibodies and their effector functions in genome-wide association studies and at the transcriptomic level in A20$^{CD11c\text{-}ko}$ mice. (**A**) The number of unique single nucleotide polymorphisms mapping to *TNFAIP3* or Fcγ receptors, according to the NHGRI-EBI GWAS Catalog. (**B**) Antibody heavy chain expression in B and plasma cells in the spleen (**C-D**) Expression levels of Fcγ receptors in wild-type (top) or A20$^{CD11c\text{-}ko}$ mice (C) or in monocytes and macrophages (D).

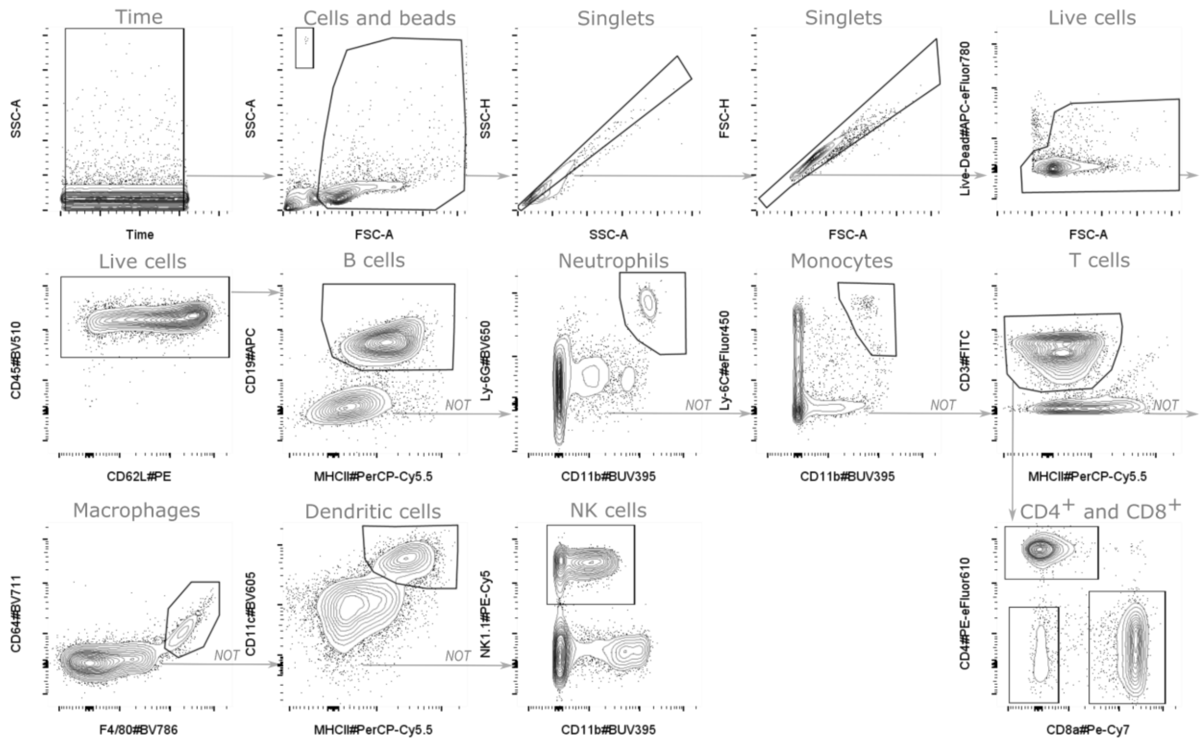

Figure S4: **Gating strategy in spleen.** A representative wild-type mouse is shown.

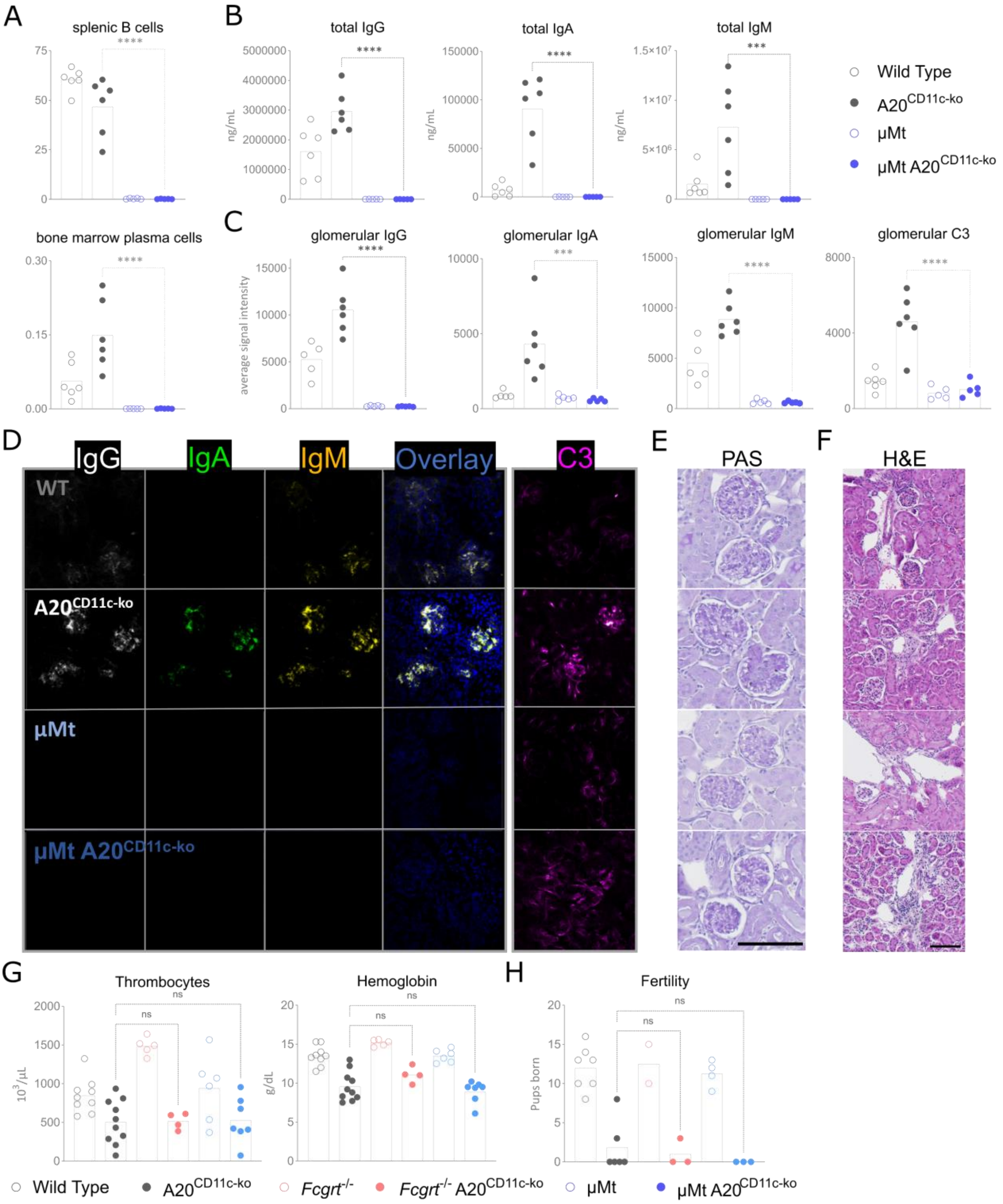

Figure S5: Effect of B cell deficiency on immune complex-deposition, hematology and fertility in A20$^{CD11c\text{-}ko}$ mice. (**A**) Quantification of B and plasma cells in spleen and bone marrow, respectively, expressed as percentage of CD45^{+} cells. (**B**) Serum antibody levels across all genotypes. (**C-D**) Glomerular immune complex and complement deposition shown as quantification (C) or representative example per genotype (D). (**E-F**) Histopathologic evaluation of glomeruli, stained with PAS (E) or H&E (F). Scale bars indicate 50 μm. (**G**) Haematological evaluation with quantification of thrombocytes (left) and haemoglobin levels (right) in peripheral blood. (**H**) Number of pups born from mothers with the respective genotype during a 10-week breeding period. Two-way ANOVAs with Šídák's multiple comparison testing were used for statistical evaluation. ns = non-significant; *** $p < 0.001$; **** $p < 0.0001$.

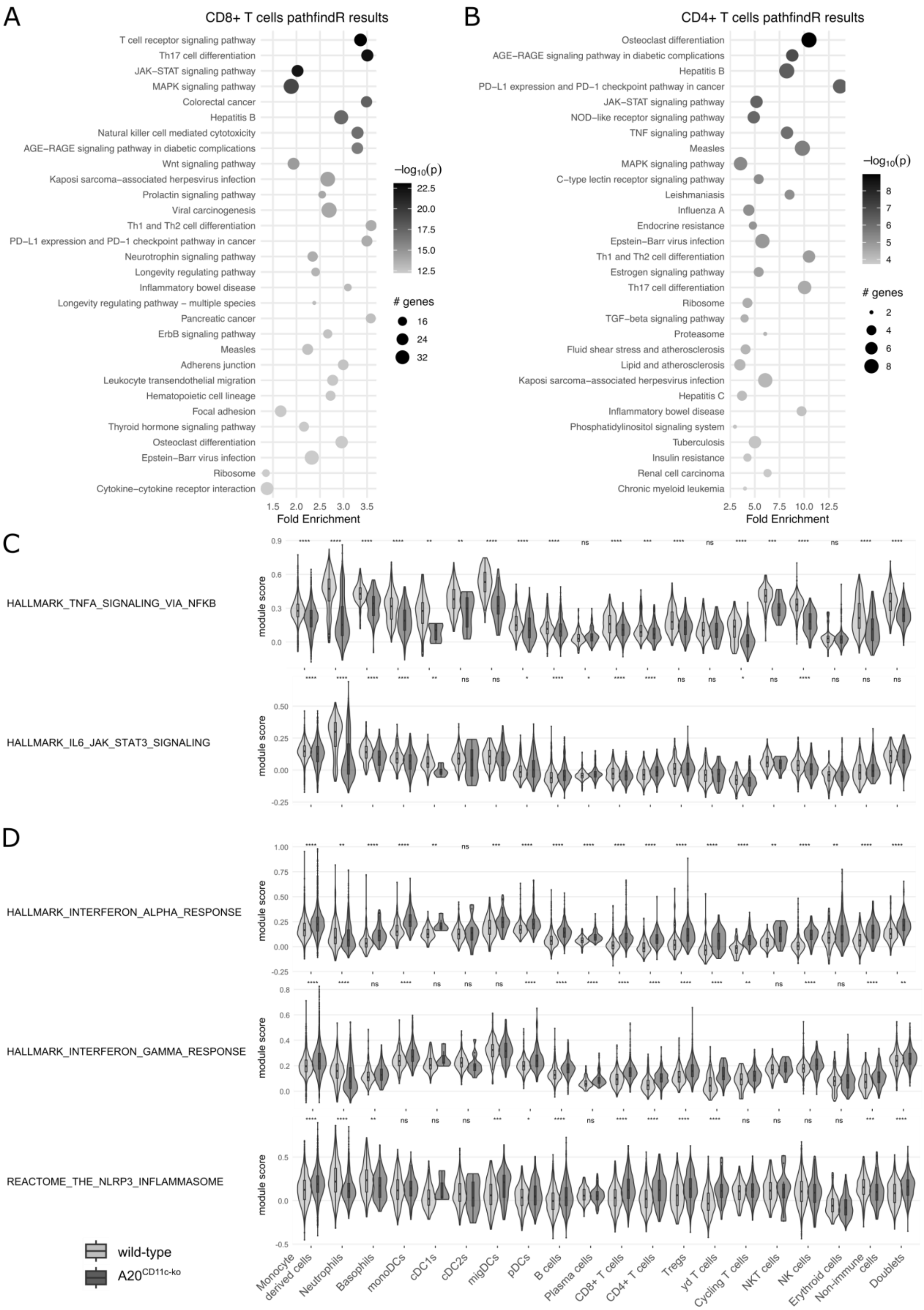
A
CD8+ T cells pathfindR results
T cell receptor signaling pathway
Th17 cell differentiation
JAK−STAT signaling pathway
MAPK signaling pathway
Colorectal cancer
Hepatitis B
Natural killer cell mediated cytotoxicity
AGE−RAGE signaling pathway in diabetic complications
Wnt signaling pathway
Kaposi sarcoma−associated herpesvirus infection
Prolactin signaling pathway
Viral carcinogenesis
Th1 and Th2 cell differentiation
PD−L1 expression and PD−1 checkpoint pathway in cancer
Neurotrophin signaling pathway
Longevity regulating pathway
Inflammatory bowel disease
Longevity regulating pathway − multiple species
Pancreatic cancer
ErbB signaling pathway
Measles
Adherens junction
Leukocyte transendothelial migration
Hematopoietic cell lineage
Focal adhesion
Thyroid hormone signaling pathway
Osteoclast differentiation
Epstein−Barr virus infection
Ribosome
Cytokine−cytokine receptor interaction
1.5 2.0 2.5 3.0 3.5
Fold Enrichment
−log10(p)
22.5 20.0 17.5 15.0 12.5
genes
16 24 32
B
CD4+ T cells pathfindR results
Osteoclast differentiation
AGE−RAGE signaling pathway in diabetic complications
Hepatitis B
PD−L1 expression and PD−1 checkpoint pathway in cancer
JAK−STAT signaling pathway
NOD−like receptor signaling pathway
TNF signaling pathway
Measles
MAPK signaling pathway
C−type lectin receptor signaling pathway
Leishmaniasis
Influenza A
Endocrine resistance
Epstein−Barr virus infection
Th1 and Th2 cell differentiation
Estrogen signaling pathway
Th17 cell differentiation
Ribosome
TGF−beta signaling pathway
Proteasome
Fluid shear stress and atherosclerosis
Lipid and atherosclerosis
Kaposi sarcoma−associated herpesvirus infection
Hepatitis C
Inflammatory bowel disease
Phosphatidylinositol signaling system
Tuberculosis
Insulin resistance
Renal cell carcinoma
Chronic myeloid leukemia
2.5 5.0 7.5 10.0 12.5
Fold Enrichment
−log10(p)
8 7 6 5 4
genes
2 4 6 8
C
HALLMARK_TNFA_SIGNALING_VIA_NFKB
HALLMARK_IL6_JAK_STAT3_SIGNALING
module score
D
HALLMARK_INTERFERON_ALPHA_RESPONSE
HALLMARK_INTERFERON_GAMMA_RESPONSE
REACTOME_THE_NLRP3_INFLAMMASOME
module score
wild-type
A20CD11c-ko
Monocyte derived cells
Neutrophils
Basophils
monoDCs
cDC1s
cDC2s
migDCs
pDCs
B cells
Plasma cells
CD8+ T cells
CD4+ T cells
Tregs
γδ T cells
Cycling T cells
NKT cells
NK cells
Erythroid cells
Non-immune cells
Doublets

Figure S6: A20-dependent immune hyperactivation arises independently of B and T cells. (**A-B**) Enrichment analysis on splenic CD8^{+} (A) and CD4^{+} T cells (B) from A20$^{CD11c\text{-}ko}$ versus wild-type mice. (**C-D**) Pathway analysis across splenic populations, more specifically TNF- and IL-6-responsive transcriptional signatures (C), type I and type II interferon signaling, as well as NLRP3 inflammasome activation (D).

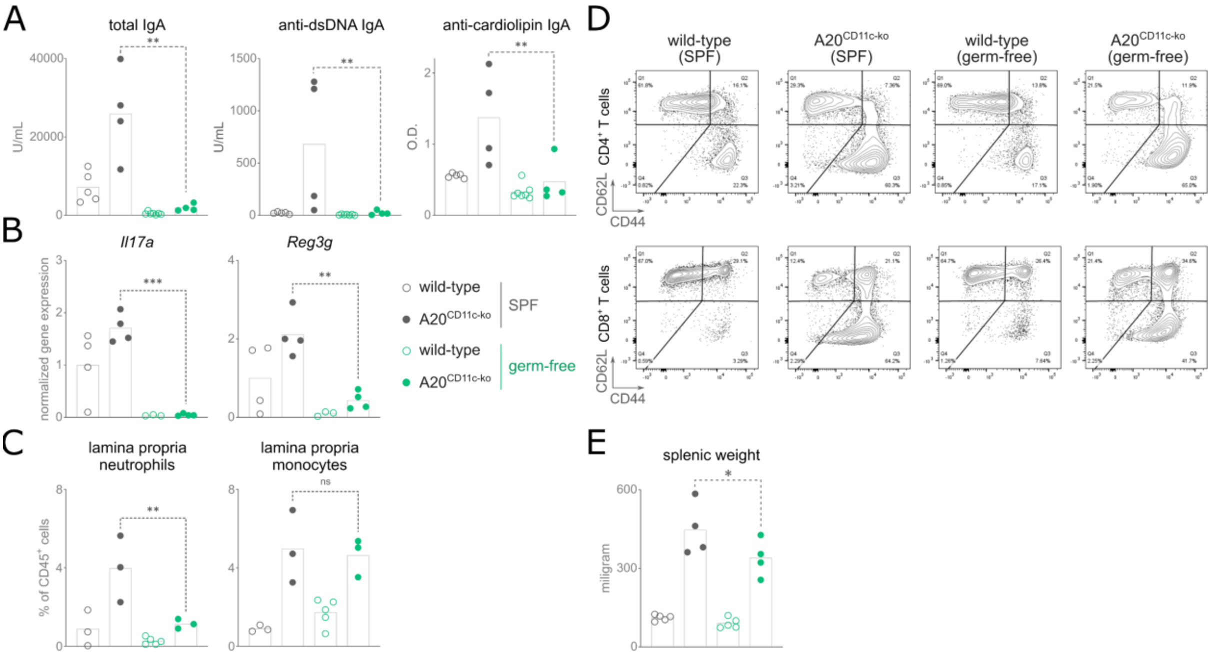

Figure S7: **Microbial influence on IgA autoantibody production and systemic inflammation in A20$^{CD11c\text{-}ko}$ mice.** (**A**) Quantification of total, anti-dsDNA, and anti-cardiolipin IgA levels in serum by enzyme-linked immunosorbent assay. O.D. = optical density. (**B**) Expression of the *Il17a* and *Reg3g*, determined by quantitative PCR on the small intestine. (**C**) Flow cytometric analysis of neutrophils and classical monocytes in the small intestine lamina propria, expressed as a percentage of total CD45^{+} cells. (**D**) Representative flow cytometry plots displaying CD62L and CD44 surface expression on CD4^{+} (top) or CD8^{+} (bottom) T cells in spleen. (**E**) Comparison of splenic weights according to genotype and microbial status. Two-way ANOVAs corrected using Šídák's multiple comparison were conducted for statistical comparisons. ns = non-significant; * $p < 0.05$; ** $p < 0.01$; *** $p < 0.001$.
